# Molecular architecture of synaptic vesicles

**DOI:** 10.1073/pnas.2407375121

**Published:** 2024-11-27

**Authors:** Uljana Kravčenko, Max Ruwolt, Jana Kroll, Artsemi Yushkevich, Martina Zenkner, Julia Ruta, Rowaa Lotfy, Erich E. Wanker, Christian Rosenmund, Fan Liu, Mikhail Kudryashev

**Affiliations:** ^a^In situ Structural Biology, Max Delbrück Center for Molecular Medicine in the Helmholtz Association (MDC), Berlin 13125, Germany; ^b^Department of Biology, Humboldt University of Berlin, Berlin, Germany; ^c^Leibniz Research Institute for Molecular Pharmacology, Berlin, Germany; ^d^Structural Biology of Membrane-Associated Processes, Max Delbrück Center for Molecular Medicine in the Helmholtz Association (MDC), Berlin, Germany; ^e^Institute of Chemistry and Biochemistry, Free University of Berlin, Berlin, Germany; ^f^Institute of Neurophysiology, Charité-Universitätsmedizin Berlin, Berlin, Germany; ^g^Department of Physics, Humboldt University of Berlin, Berlin, Germany; ^h^Neuroproteomics, Max Delbrück Center for Molecular Medicine in the Helmholtz Association (MDC), Berlin, Germany; ^i^Institute of Pharmacy, Free University of Berlin, Berlin, Germany; ^j^Charité-Universitätsmedizin Berlin, Berlin, Germany; ^k^Institute of Medical Physics and Biophysics, Charité-Universitätsmedizin Berlin, Berlin, Germany

**Keywords:** synaptic vesicles, V-ATPase, clathrin, cryo-ET

## Abstract

Synaptic vesicles (SVs) store and transport neurotransmitters to the presynaptic active zone for release by exocytosis. After release, SV proteins and excess membrane are recycled via endocytosis, and new SVs can be formed in a clathrin-dependent manner. This process maintains complex molecular composition of SVs through multiple recycling rounds. Previous studies explored the molecular composition of SVs through proteomic analysis and fluorescent microscopy, proposing a model for an average SV (1). However, the structural heterogeneity and molecular architecture of individual SVs are not well described. Here, we used cryoelectron tomography to visualize molecular details of SVs isolated from mouse brains and inside cultured neurons. We describe several classes of small proteins on the SV surface and long proteinaceous densities inside SVs. We identified V-ATPases, determined a structure using subtomogram averaging, and showed them forming a complex with the membrane-embedded protein synaptophysin (Syp). Our bioluminescence assay revealed pairwise interactions between vesicle-associated membrane protein 2 and Syp and V-ATPase Voe1 domains. Interestingly, V-ATPases were randomly distributed on the surface of SVs irrespective of vesicle size. A subpopulation of isolated vesicles and vesicles inside neurons contained a partially assembled clathrin coat with an icosahedral symmetry. We observed V-ATPases under clathrin cages in several isolated clathrin-coated vesicles (CCVs). Additionally, from isolated SV preparations and within hippocampal neurons we identified clathrin baskets without vesicles. We determined their and CCVs preferential location in proximity to the cell membrane. Our analysis advances the understanding of individual SVs' diversity and their molecular architecture.

## Significance

In this study, Kravčenko and colleagues advance our understanding of synaptic vesicles (SVs), crucial for neurotransmitter storage and release. Employing cryoelectron tomography, the study characterizes a diversity of SV proteins, including small proteins on the SV surface, elongated proteins inside, and large V-ATPases randomly distributed on the surface of SVs. V-ATPase structure revealed an additional transmembrane interaction partner synaptophysin. The study uncovers V-ATPases under clathrin cages of clathrin-coated vesicles and partially assembled clathrin coats on vesicles ex vivo and within neurons, providing insights into their structural symmetry. Furthermore, the study identifies clathrin baskets without vesicles near the cell membrane. These findings highlight the intricate molecular architecture of SVs, offering a broad perspective and complementing traditional proteomic analysis and fluorescent microscopy.

Neuronal communication is based on the coordinated and tightly regulated fusion of synaptic vesicles (SVs) with the presynaptic membrane and the release of neurotransmitters (NT) into the synaptic cleft. Previous studies demonstrated that the surface of SVs is crowded with more than 40 different proteins involved in NT release and SV recycling (1–3). The most abundant proteins are SNAREs (VAMPs, syntaxins), calcium sensors (synaptotagmins), endocytosis-related proteins (dynamins), small GTPases (Rabs), and other trafficking and membrane proteins (CSP) (2, 4). While the molecular composition of SVs has been well studied using mass-spectrometry (1, 2, 5, 6) and fluorescence microscopy (3, 7), the architecture of individual SVs has not been analyzed at molecular resolution.

Neuronal depolarization induces the fusion of NT-filled SVs with the presynaptic active zone membrane, leading to the release of their contents into the synaptic cleft and signal propagation to the postsynapse (8). During subsequent endocytosis, SV proteins and excessive membranes are internalized and new SVs are reformed. To preserve the molecular composition, size, and shape of SVs throughout several rounds of their fusion and recycling, these processes must be tightly controlled (9). To couple exo- and endocytosis and to organize the proper reformation of SVs, highly abundant SV proteins vesicle-associated membrane protein 2 (VAMP2) and Syp interact with the clathrin adaptor AP180 and the GTPase dynamin at the periactive zone, respectively (10, 11). While the endocytic internalization of membranes can be clathrin-independent, e.g. via ultrafast endocytosis or activity-dependent bulk endocytosis, the reformation of new SVs was shown to be clathrin-dependent (12). It is likely that the organization of clathrin triskelia in pentamers and hexamers is required to produce membrane curvature and lead to the formation of vesicles with a defined size (13, 14). Clathrin-coated vesicles (CCVs) were shown to have several symmetry types defined mostly by the number and arrangement of pentagonal or hexagonal faces, which are organized by the adaptor proteins (15). Interestingly, also non-vesicle-carrying clathrin cages (baskets) were observed to be formed in vitro and hypothesized to be assembled spontaneously from monomers during the isolation procedure (16, 17).

To reform fusion-competent SVs, CCVs must be uncoated, their lumen needs to be acidified via V-ATPases and NT must be loaded via transporters like VGLUT, VMAT, VAChT, and others. Short before SV fusion, the extravesicular V1 region of the V-ATPase, required for ATP hydrolysis, is dissociated from the membrane-embedded Vo region (18). After SV fusion, the V1 region is recruited back to the SV, likely via rabconnectin-3 (19). Although immunoblot experiments have suggested that CCVs contain both Vo and V1 regions (20), it is unclear whether the two domains are already assembled on CCVs. In contrast to the V1 region, the Vo region remains integrated into the membrane during fusion and is recycled during endocytosis. Considering that each SV has on average 1.4 V-ATPases (1), it is conceivable that the tight control of the Vo region recycling and the V1 region recruitment are crucial for the proper timing of fast SV refilling, particularly during sustained NT release.

In the present study, we performed cryoelectron tomography (cryo-ET) on neuronal SVs and CCVs within cultured hippocampal mouse neurons in situ and isolated vesicles from mouse brains ex vivo to characterize their molecular architecture. This enabled us to categorize different types of proteins visible at the SV surface according to their shape and size. Beyond that, subtomogram averaging (StA) revealed a structure of the V-ATPase interacting with a small transmembrane protein that we identified as Syp. We observed assembled V-ATPases under the cage of CCVs, and characterized partially (un)coated CCVs both in situ and in ex vivo preparations. Interestingly, we detected empty clathrin cages, performed StA on them, and characterized their size, type of symmetry, and preferred cellular localization.

## Results

### Cryo-ET of SVs from Primary Hippocampal Neuron Cultures in Situ and Isolated from Mouse Brains.

**Fig. 1.**
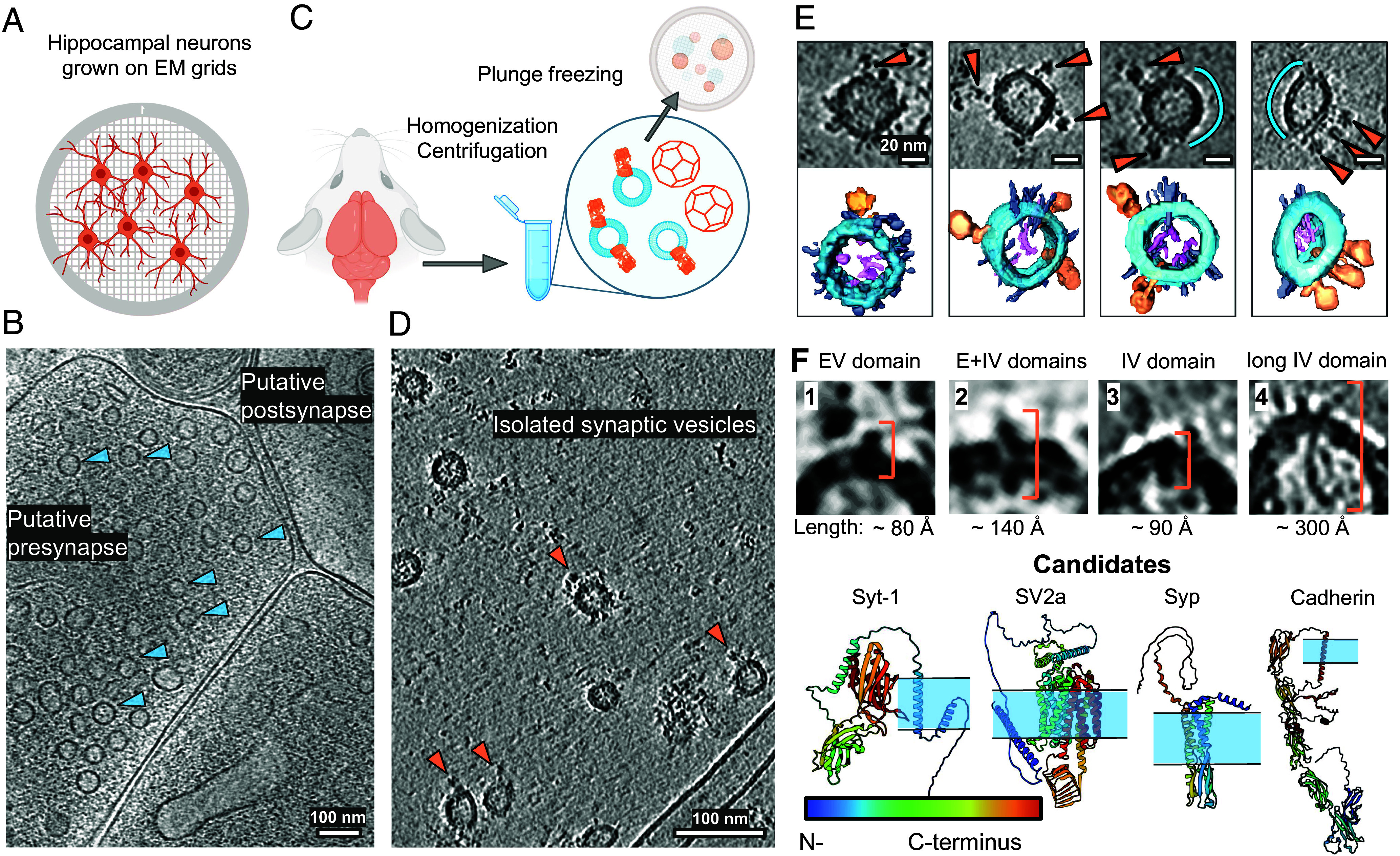
Visualization of individual SVs and proteins on their surface. (*A*) Schematic representation of primary hippocampal neurons grown on grids. (*B*) A slice through a cryo-ET volume of a putative presynapse in situ containing individual SVs (shown with blue arrows). (*C*) Schematic representation of SV isolation procedure. (*D*) A slice through a cryo-ET volume of isolated SVs. The tomogram was processed by IsoNet (21). (*E*) Upper row: isolated individual SVs, with proteins on their surface. Blue curves indicate SV surfaces without detectable proteins. Lower row: segmentations showing V-ATPase densities (orange), intravesicular protrusions (pink), and small membrane proteins (gray) on the surface of SVs (blue). (*F*) Protein densities are grouped into classes based on their shape, size, and membrane topology in the tomograms of isolated SVs. Predictions of structures of potential candidates for observed densities from the AlphaFold2 (22) database and a scheme of their membrane localization (the membrane is schematically shown in blue). N to C termini for framed AlphaFold2-predicted proteins are shown from blue to red. V-ATPase PDB: 6wm2 (23). (Scale bars: (*B*), (*D*) 100 nm, (*E*) 20 nm.) V-ATPases are shown with orange arrows in (*D*) and (*E*).

We first aimed to visualize the composition of SVs in situ. For this, we imaged presynaptic areas of mouse hippocampal neurons cultured on electron microscopy grids (DIV 17) by cryo-ET (Fig. 1*A*). We recorded 88 tomograms containing 56 putative presynaptic terminals (Fig. 1*B* and *SI Appendix*, Table S1). The tomograms showed expected synaptic topology and did not contain signs of cellular stress. In order to obtain tomograms with higher signal, we extracted vesicles from mouse brain tissue (hippocampus, cortex, and cerebellum) (Fig. 1*C*). After confirming successful vesicle purification via mass-spectrometry (Dataset S1), we recorded 719 tomograms containing vesicles purified from mouse brains (*Methods,* *SI Appendix*, Table S1). These tomograms of isolated vesicles provided a higher signal-to-noise ratio and allowed visualization of the molecules on their surface in more detail (Fig. 1*D*). To preserve the molecular architecture of vesicles as well as possible, we chose a gentle purification protocol with only two centrifugation steps and without affinity columns (*Methods*). As a consequence, our vesicle preparation also contained fractions of other classes of vesicles like CCVs. The two datasets are complementary, as the in situ data allows more native preservation, while the tomograms of purified vesicles provide more contrast for molecular identification.

The in situ dataset was originally prepared for a study in which neurons were optogenetically stimulated a few ms before cryofixation. A first stimulus was applied 100 ms before freezing, a second one 2 to 5 ms before freezing, each of them inducing a single action potential (for details, see *Methods*). We compared tomograms from the optogenetically stimulated grids with an unstimulated control sample frozen under the same conditions to make sure that the optogenetic stimulation did not influence the size, shape, and distribution of SVs and CCVs. As we did not observe any differences between unstimulated and optogenetically stimulated samples, we analyzed those data together and refer to them as in situ dataset.

### Molecular Landscape of Synaptic Vesicle Membrane.

In both datasets — in situ and in purified fractions — we first characterized SVs without clathrin coat. Due to the potential heterogeneity of vesicle populations in our preparation, we only considered SVs containing at least one V-ATPase for our analysis, and we possibly missed the vesicles containing only Vo subunit (3,529 SVs, Dataset S2). Characteristic protein densities particularly on the surface of SVs but also within the SV lumen were visible in both preparations (Fig. 1 *B* and *D* and *SI Appendix*, Fig. S1). Since the resolution of tomograms of isolated SVs was higher than that of SVs in situ due to the lower thickness of vitreous ice, we focused on isolated SVs for protein identification and characterization. Interestingly, we could observe that while most of the surface of SVs was covered by protein densities, some areas did not have apparent proteins (Fig. 1*E*, empty surface is shown with blue segments). We next classified the apparent protein densities into four structural classes, according to their size and membrane topology: 1) small extravesicular (EV) domain; 2) extra-and-intravesicular (E + IV) domains; 3) small intravesicular (IV) domain; and 4) long IV domain (Fig. 1*F*).

We aimed to quantify the abundance of proteins from different classes per SV. For this, five independent expert annotators who did not know the previously reported proteomic composition (1, 5), inspected 90 SVs to identify protein-like densities and assign each observed density to one of the four suggested structural classes. To estimate the abundance of proteins from outlined classes, the mean value of expert counts per each structural class was calculated for each SV individually, followed by averaging the obtained mean counts per class over all SVs. Quantified occurrences of smaller proteins per SV were statistically unreliable due to their size. However, our calculations showed an average number of long IV protrusions from class 4 per SV (90 SVs per expert annotator) to be 1.0, with some vesicles having up to 4 (*SI Appendix*, Fig. S1 *A* and *B*). Long proteins were observed in 59% of the analyzed SVs. Due to their flexibility, we could not average them using StA (24, 25). The length of protrusions was between ~120 to 400 Å, with the most abundant value of ~180 Å. For some of them, we observed an EV (cytoplasmic) part (Fig. 1*F* and *SI Appendix*, Fig. S1 *A* and *B*).

To match the potential molecular identities for our morphologically defined protein classes 1 to 4, we examined AlphaFold2 (22) predictions for the proteins previously reported to be present in SVs (1, 5) (Fig. 1*F* and *SI Appendix*, Table S2–S4). UniProt (26) annotation of the AlphaFold2 (22) predictions, containing the information about their lumenal, transmembrane, and cytoplasmic domains, allowed us to categorize and match them with corresponding densities observed in tomograms regarding their shape and membrane topology. For example, the SV protein SV2a (27) contains relatively large intra- and EV domains, resulting in the noticeable density present on both membrane sides, making it a candidate for class 2. On the contrary, Syp (28) contains a relatively large IV domain and a transmembrane domain, but no appreciable EV domain, being a candidate for class 3 (Fig. 1*F*). The set of proteins predicted as having only an EV domain or both EV and transmembrane domains are synaptotagmins, Rab3A, synapsins, RalA, SEC22b, SCAMPs (class 1); class 2 resembles SV2 proteins (SV2a, SV2b, SV2c), class 3 resembles Syp, synaptogyrins (Syngr1, Syngr3), Syp-like protein 1 (Sypl1), and synaptoporin (Synpr) (Fig. 1*F* and *SI Appendix*, Table S2–S4).

To identify potential proteins inside the vesicles, we performed a proteinase K assay (29). In this assay, EV domains of proteins are digested by proteinase K and only proteins located within the membrane or vesicle lumen are protected. The ratio of protected proteins was quantified by label-free mass-spectrometry (Dataset S3 and *SI Appendix*, Fig. S2), showing most of the proteins to be proteinase K sensitive. Our analysis of the predicted structures from the SV proteome did not reveal candidates for class 4. Therefore, we examined a list of earlier described proteins with extracellular domains that are typically located close to synaptic release sites: cadherins (30), neurexins, and neuroligins (31). Among them, N-Cadherin was the most similar candidate according to its size and shape. We analyzed the AlphaFold2 predictions for N-Cadherin (~225 Å in length) (30), which are known to mediate cell–cell adhesion, cover the axon terminal, and undergo constant endocytosis (32, 33). Cadherins have five tandem repeating extracellular domains, a transmembrane domain, and a smaller intracellular domain (30). Cadherin-2, 6, 10, 11, and 13 were found in our proteomics analysis (Dataset S1), and cadherin 2 and 13 were protected in our proteinase K assay (*SI Appendix*, Fig. S2 *C, D* and Dataset S3). Considering the size of the density and our proteomics analysis, we hypothesize that a fraction of the observed intravesicular densities could be synaptic adhesion molecules like cadherins (*SI Appendix*, Table S5).

### The V-ATPase Forms a Complex with Syp.

**Fig. 2.**
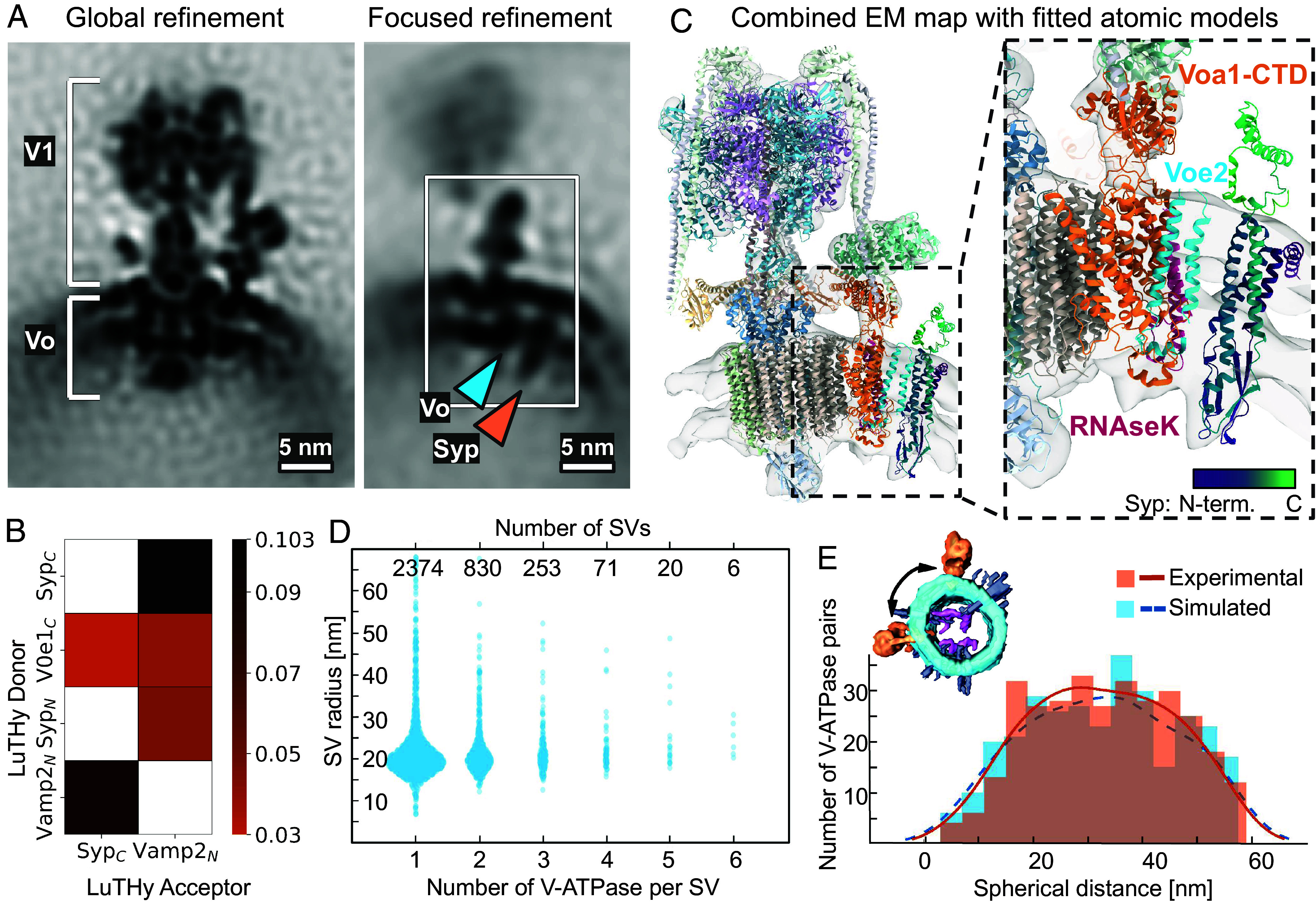
V-ATPase-Syp complex and V-ATPases arrangement on the surface of SVs. (*A*) *Left*: structure of V-ATPase on the SV’s surface at 16.7 Å resolution. *Right*: An extra density (marked with an orange arrow) near the V-ATPase Vo region (marked with a blue arrow). (Scale bars, 5 nm.) (*B*) Selected interactions (BRET signals) obtained with LuTHy assays performed with V-ATPase Voe1 domain, Syp, and VAMP2. Control-corrected BRET (cBRET) ratios for the tested pairs of interacting partners as the heatmap, representing protein interaction strength with a color gradient from orange to black. Only interactions with observed cBRET values ≥0.03 (cutoff for membrane proteins) are shown. Nontested pairs are shown by noncolored/white cells. (*C*) Rigid-body fit of the V-ATPase and Syp atomic models into the combined map, obtained by merging global and focus-refined StA maps of V-ATPase from isolated SVs. V-ATPase Voe2 domain is shown in blue, the Voa1-CTD domain is shown in orange, RNAseK domain is shown in magenta. Syp is shown in viridis (violet-to-green) gradient, corresponding to the N-to-C termini. An atomic model of a purified V-ATPase complex (PDB: 6wm2) and an AlphaFold3 (34) prediction of Vo domains (a1, e2, RNAseK) in complex with Syp (UniProt ID: Q62277) were used (*Methods*). Visualization was performed in ChimeraX (35). (*D*) Correlation of SV radii distributions, with SVs being categorized by the number of V-ATPases identified on their surfaces. (*E*) Experimental (orange, continuous) and simulated (blue, dashed) distributions of spherical (geodesic) distances between pairs of V-ATPases on SV surfaces containing two V-ATPases. The measured distance is schematically represented with a black arrow at the volume-rendered SV.

We next identified V-ATPases in our tomograms and generated a structure at a resolution of 16.7 Å from 5361 particles by StA (Fig. 2*A**, Left,* *SI Appendix*, Fig. S3). A previously reported atomic model of human V-ATPase from single-particle cryo-EM [PDB: 6wm2 (23)] fitted well into the density. The limited number of particles without symmetry and the highly dynamic structure of the V-ATPase likely were the main resolution-limiting factors. Interestingly, in our V-ATPase structure, we observed an additional membrane-embedded density that was not accommodated by the atomic model of the V-ATPase. The density had a small SV-lumenal domain, located a few nanometers away from the Vo-a1-e2-RNAseK-proximal region. A refinement focused on this area resulted in a better-defined density of ~65 Å in length with a resolution of 21.1 Å (Fig. 2*A**, Right,* *SI Appendix*, Fig. S3). Focused classification without alignment also showed that all resulting classes have this density.

To probe potential candidates for this density, we employed an integrative structural biology approach. We analyzed the proteins from classes 2 and 3 (SV2a, SV2b, SV2c, Syp, Synpr, Syngr1, Syngr3, and Sypl1) for the presence and abundance per SV in the reported (1, 5) and our proteomics data (Dataset S1), stability to the proteinase K treatment (our data, Dataset S3) and reported (36) and predicted protein–protein interactions (PPI) (*SI Appendix*, Fig. S4). We predicted the atomic models of complexes of Voe2, Voa1, RNAseK, and candidate proteins by AlphaFold3 (AF3) (34) (*SI Appendix*, *SI Materials and Methods*). We fitted candidate protein’s atomic models into the StA density and calculated the fitting score (*SI Appendix*, *SI Materials and Methods*). Although the model’s interaction and StA density fitting scores were the highest for three candidates Syp, Sypl1, and Synpr (structurally similar to Syp), all provided integrative data evaluation for the candidate proteins showed Syp to be the top candidate (Table 1).

**Table 1.** Evaluation of the possible candidates for the interaction with the Vo subunit for the proteins from classes 2 and 3

Candidate protein, UniProt ID	Syp, Q62277	Synpr, Q8BGN8	Syngr1, O55100	Syngr3, Q8R191	Sypl1, O09117	SV2a, Q9JIS5	SV2b, Q8BG39	SV2c, Q69ZS6
Presence in MS data (Wittig et al.(5))	+	+	+	+	–	+	+	+
Presence in MS data (Takamori et al.(1))	+	–	+	+	–	SV2: +		
Presence in our MS data	+	+	+	+	(+)^*^	+	+	+^1^
Amount per SV (Takamori et al.(1))	31.5	Not available	Synaptogyrin: 2		0	SV2: 1.7		
Amount per SV (Wittig et al.(5))^**^	15.5	1.4	10	4.6	0	4.6	2.6	0.1
Proteinase K resistance	+	–	–	–	Not available	–	–	–
MS cross-link with Vo-V-ATPase (untreated SVs) (Wittig et al.(5))	+	–	–	Not available	Not available	–	–	Not available
Interaction with V-ATPase Vo (Galli et al. (36))	+	Not available	Not available	Not available	Not available	Not available	Not available	Not available
Interaction with V-ATPase AF3 ipTM_prot_^***^	0.32	0.26	0.15	0.21	0.30	0.27	0.18	0.24
Fit to Vo-proximal (focused StA map) score	0.82	0.83	0.78	0.78	0.86	0.78	0.62	0.53
Sequence Identity to Syp^****^	100%	56%	8%	14%	46%	10%	7%	5%
TM-score to Syp^****^	1.0	0.67	0.44	0.45	0.65	0.31	0.32	0.32

^*^The protein detection yielded an iBAQ value of 0.000007, indicating an extremely low abundance (Dataset S1).

^**^The value was calculated using Wittig et al.’s *SI Appendix*, Table S1 (Protein identification in SV), which provides relative iBAQs for identified proteins. To get a number of each protein from the table per SV, each protein’s relative iBAQ (37) was divided by the V-ATPase-A relative iBAQ, then multiplied by 3 (number of A subunits per V-ATPase) and 1.4 (previously reported average number of V-ATPases per SV1).

^***^The protein-only chain-to-all ipTM AF3 prediction quality metric for lipids-inclusive complexes predictions was used (*SI Appendix*, *SI Materials and Methods*).

^****^PDB Pairwise Structure Alignment server (www.rcsb.org/alignment).

To experimentally test whether Syp interacts with subunits of the V-ATPase, we next performed a quantitative evaluation of PPI by the luminescence‐based two‐hybrid assay LuTHy (38). LuTHy identifies direct interactions between tagged fusion proteins expressed in mammalian cells by quantification of bioluminescence resonance energy transfer (BRET) (Fig. 2*B*, *SI Appendix*, Fig. S5 and Dataset S4). The assay demonstrated interactions between the C terminus of human Syp and the C terminus of the human Voe1, an isoform of which was shown to be a subunit of the V-ATPase (23). Furthermore, the single transmembrane helix-protein VAMP2 interacted with both Syp and the Voe1 subunit of the V-ATPase (Fig. 2*B*, *SI Appendix*, Fig. S5 and Dataset S4). VAMP2 has a low molecular weight, a single transmembrane helix, and its positioning by Alphafold3 had low scores, therefore we could not reliably model it. However, our integrative structural analysis leads to the conclusion that the additional density in the V-ATPase is accommodated by Syp, in agreement with the recent cryo-EM reports (39, 40).

### Distribution of V-ATPases on the Surface of SVs.

Some V-ATPases were observed to form apparent clusters on SVs, while others seemed to be positioned away from each other (Fig. 1*E*). To quantify the distribution of V-ATPases, we manually confirmed the identity of V-ATPases using their positions and orientations obtained by StA. We matched the V-ATPases and the SVs and determined the radius of the corresponding SVs with a set of algorithms (*SI Appendix*, SI Materials and Methods). In brief, each of 5361 V-ATPases in the final set of true particles was assigned to an SV and the radius of the corresponding SV was automatically estimated, using the orientation of the V-ATPases (*SI Appendix*, *SI Materials and Methods*). V-ATPases on the vesicles with the same centers were counted. From 3554 identified SVs, 63% contained one V-ATPase/SV, 26% two V-ATPases/SV, 8% three V-ATPases/SV, and the minority contained more than three V-ATPases/SV (Fig. 2*D*). The average number of V-ATPases per SV in this analysis is 1.47, closely matching the reported value of 1.4 (1). Surprisingly, we found that the number of V-ATPases per SV does not correlate with the SV radius (Pearson correlation coefficient r = -0.0025, *P* = 0.894 > 0.05, n = 3554).

To quantitatively characterize the mutual arrangement of V-ATPases on the surface of SVs, we calculated the distance between the centers of V-ATPases across the membrane, referred to as the “spherical distance” (Fig. 2*E*). For this, we only analyzed SVs with radii of 19 or 20 nm and two V-ATPases present on their surfaces (n = 320 SVs). Despite the nonuniform distribution profile, its rather broad peak (~20 to 45 nm) appearance did not show any preferred distances between particles, while its nonuniformity is caused by the orientation bias in particle picking: V-ATPases, which are oriented in the direction of the electron beam, are underrepresented due to the “missing wedge” effect, a distortion associated with the cryo-ET imaging procedure causing an anisotropic resolution. To evaluate this, we simulated a random distribution of paired particles on the surface of SVs with the given radius, imposing the particle orientations bias observed for our V-ATPase dataset (*SI Appendix*, *SI Materials and Methods*). The distribution of pairwise distances reflected a nonuniform profile, similar to the experimental data, due to the accounted underrepresentation of the "top views" of V-ATPases (Fig. 2*E*). The statistically equal distribution of measured and simulated pairwise distances (*P* = 0.44, two-sample Kolmogorov–Smirnov nonparametric statistical test) indicates that V-ATPases are randomly distributed on the surface of SVs without any specific clustering mechanism. We applied the same analysis to SVs with three V-ATPases and found no difference in the distributions.

### Some CCVs Are Not Fully Covered.

**Fig. 3.**
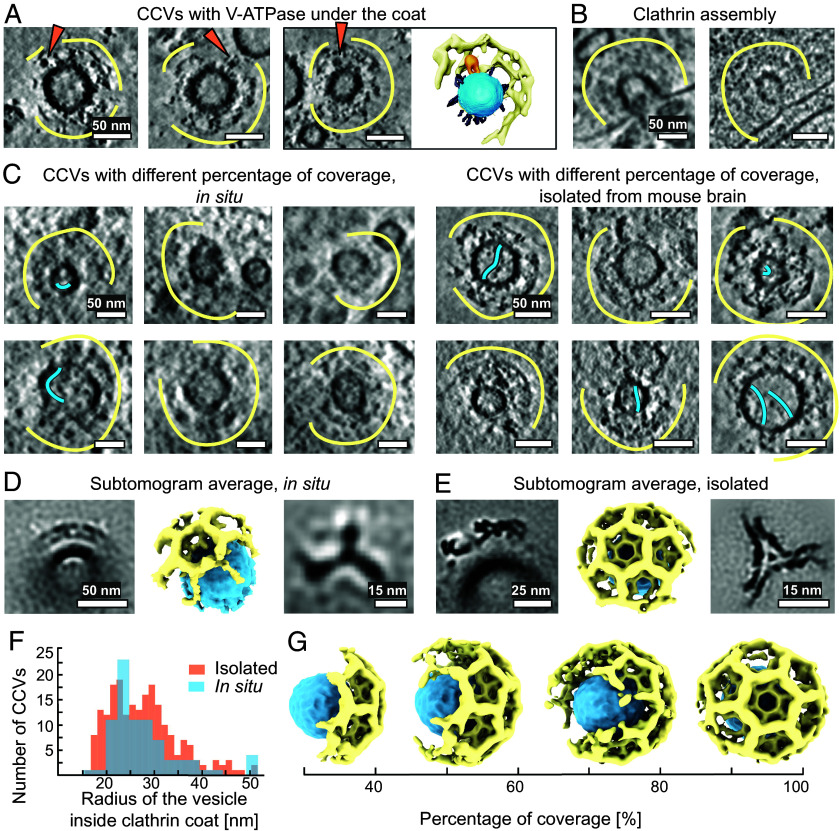
Distribution of clathrin on the surface of vesicles. (*A*) CCVs contain V-ATPase under their cages and a segmentation of one of them. V-ATPases are shown with orange arrows, small proteins in gray, clathrin cage — with yellow curves. Segmentation: The vesicle membrane is blue, V-ATPase is orange, clathrin cage — yellow. For clarity, only half of a clathrin coat is segmented. (Scale bar is 50, nm.) (*B*) In situ CCVs in the process of assembly on the membrane. (Scale bar, 50 nm.) (*C*) *Left*: CCVs from primary hippocampal neurons, with different percentages of clathrin coverage. Clathrin cages are shown with yellow curves. Long IV protrusions are shown with blue curves. *Right*: CCVs, isolated from mouse brain tissue, with different percentages of clathrin coverage. (Scale bars, 50 nm.) (*D*) StA of CCV fragment from primary hippocampal neurons (*Left*, Scale bar, 50 nm) with its volume rendering (ChimeraX, center) and of a single clathrin triskelion (*Right*, Scale bar, 15 nm.) (*E*) StA of CCV fragments, isolated from mouse brain tissue (*Left*, Scale bar, 25 nm) with its volume rendering (ChimeraX, center) and of a clathrin triskelion (*Right*, Scale bar, 15 nm.) (*F*) Histograms of CCVs radii (CC-enclosed vesicle center-to-membrane distances) distribution, isolated from mouse brain tissue (orange, bin size = 1.34 nm) and observed in neurons (blue, bin size = 2.45 nm). G) Volume-rendered representations of CCVs, illustrating the different percentages of coverage with clathrin.

We observed fully assembled V-ATPases not only at SVs but also under clathrin cages in the ex vivo preparation (Fig. 3*A*). As the V1 domain is dissociated from the Vo domain shortly before fusion (18), we propose that the reassembly of the V1 domain to the Vo domain after SV fusion can happen early during endocytosis. More precisely, the recruitment may occur prior to the complete assembly of the clathrin cage around the newly reformed SV, rather than during or after the uncoating step. The V1 heads were observed under the layer of the clathrin cage, as would be expected from the V1 region height (~19 nm) and the height between the vesicle membrane and clathrin triskelion (~24 nm, *SI Appendix*, Fig. S6).

Next, we investigated CCVs in tomograms from in situ neuronal terminals and in tomograms of isolated vesicles. We found a few examples of clathrin-coated pits (CCPs) at the cell membranes of neurons with an open or already constricted neck (Fig. 3*B* and *SI Appendix*, Fig. S7 *A*). Due to the small number of CCPs, we did not characterize them further but focused on CCVs already fissioned from the membrane or endosomes. While previous studies investigated clathrin symmetry variation and cage size in CCVs purified from brain tissue (15, 16) or assembled in vitro (13, 14, 17, 41), we reexamined their size, clathrin coverage, and cage symmetry in purified vesicles and inside primary hippocampal neurons using cryo-ET. We attempted the quantification of coverage of CCVs with clathrin in the purified vesicle sample by subtomogram classification. Due to heterogeneity, the presence of a missing wedge, and limited signal, subtomogram classification underestimated the coverage, therefore we assessed it qualitatively. By manual visual analysis, we found that 45% of the examined CCVs in the isolated vesicle sample contained a disrupted clathrin coat in the missing-wedge-free direction (n = 172). The vesicles from this dataset were isolated without adding ATP, which is required for Hsc70-mediated CCV uncoating (42). In addition, we examined isolated vesicles in the presence of ATP and found that here, 85% of the CCVs had an incomplete clathrin coat (n = 40). This confirms that ATP is relevant for clathrin disassembly and demonstrates that our relatively gentle purification allowed for the coordinated and protein-mediated disassembly of clathrin coats. We further assessed CCVs in situ, whereby 31% of the examined CCVs were found at the presynapse, 31% at the postsynapse, and 38% in extrasynaptic compartments. In the in situ dataset, 37% of CCVs, independent of their cellular localization, contained only partial clathrin coats in the missing-wedge-free direction (Fig. 3*C* and *D* and *SI Appendix*, Fig. S7 A), however, the disruptions of clathrin coats are harder to detect in the in situ tomograms due to the lower signal-to-noise ratio and the more crowded cellular environment. As the noncoated SVs, some CCVs also contained long IV protrusions (Fig. 3*C*, *SI Appendix*, Fig. *S1 C* and *D**)*.

The knowledge of the shape and symmetry of the clathrin coat is essential for elucidating its function and regulation (15, 17). Therefore we tested the predominant arrangement of clathrin on the surface of the CCVs in situ and isolated vesicles. For CCVs (all vesicles containing a partial or complete clathrin coat, with or without visible V-ATPases) we picked 81 CCVs from tomograms of hippocampal neurons, and 212 CCVs from purified fractions (Fig. 3*C*). Here, we were selective in the quality of tomograms, especially preferring thinner cellular tomograms. Nonetheless, such numbers were sufficient to obtain moderate resolution structures. For our in situ data, we did not only select CCVs at the presynapse but also CCVs in less crowded environments like axonal regions, whereby we did not notice any structural differences between different locations. Parameterization of cellular CCVs surface and subtomogram classification and averaging, without the application of symmetry (C1) resulted in a low-resolution StA structure of a clathrin coat fragment at 76 Å resolution, containing 6 triskelia (Fig. 3*D* and *SI Appendix*, Fig. S3). Most of the vesicles inside clathrin cages had radii of 25 nm (Fig. 3*F* and Dataset S5).

We next structurally analyzed 212 CCVs from the purified vesicle fraction (Fig. 3*C*, *Right*). The distribution of CCV outer membrane radii showed two peaks: at 23 nm and 31 nm (Fig. 3*F*). The observed variability of radii is likely due to the origin of the CCVs: while the vesicles from the first radii peak are possibly from synapses, the larger vesicles may be related to other cellular processes. The difference between the first peak for the purified vesicles (23 nm) and the main peak in situ (25 nm) is about 1 pixel and is within the precision of measurement. We performed StA of the clathrin lattice containing 20 triskelia in the alignment mask, resulting in a structure at a resolution of 27 Å (Fig. 3*E* and *SI Appendix*, Fig. S3). The structure showed a hexamer surrounded by 3 pentamers and 3 hexamers (icosahedral cage symmetry) (Fig. 3*E*). Separate processing of the vesicles with smaller and larger radii showed that they have the same icosahedral symmetry (*SI Appendix*, Fig. S7*B*). Thus, in both isolated CCVs and vesicles in situ, we observed an icosahedral symmetry as the most dominant type of clathrin arrangement, independent of the vesicle radii.

### Synapses Contain Clathrin Baskets Without Vesicles Inside.

**Fig. 4.**
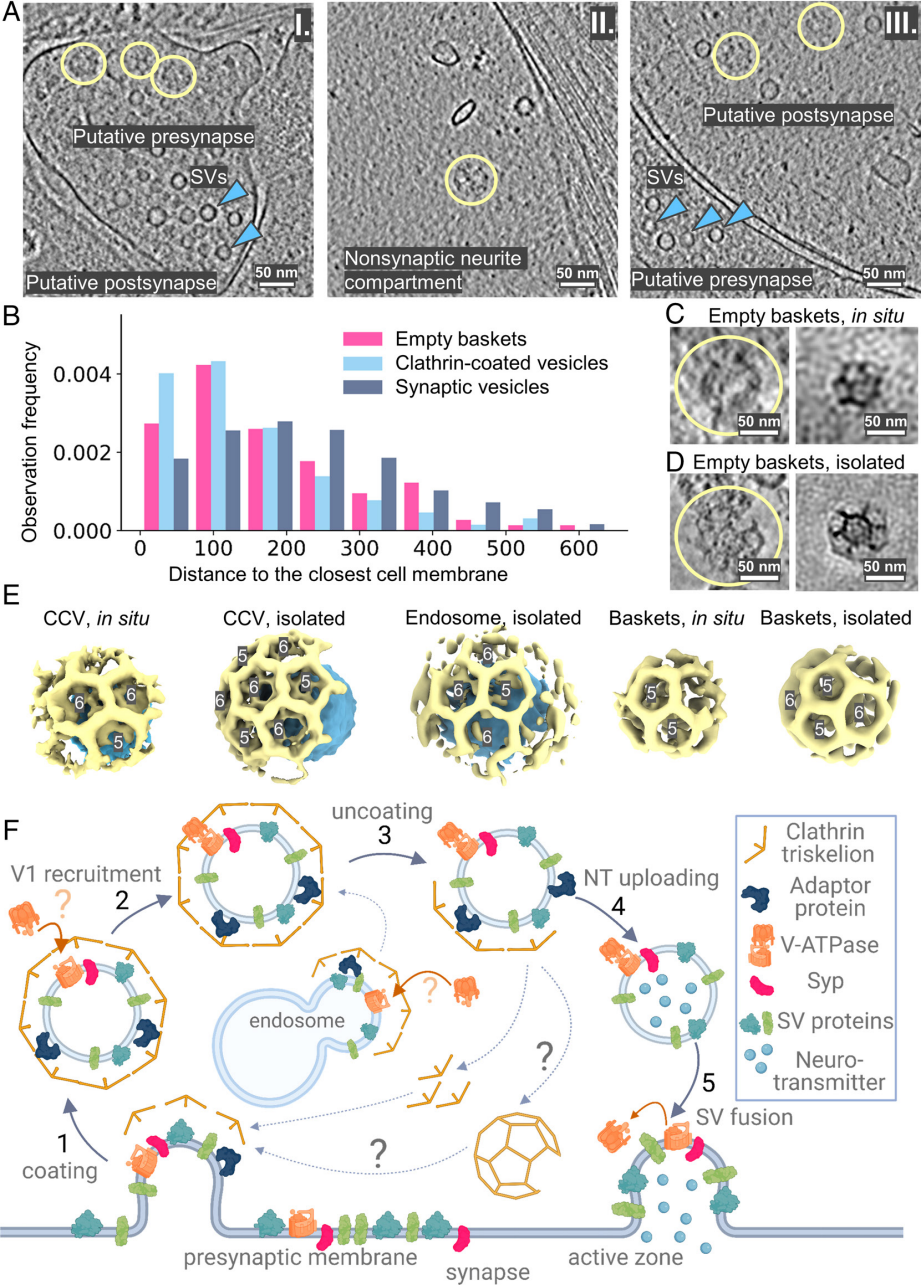
Non-vesicle-containing clathrin baskets are present in cells. (*A*) Slices through tomograms with empty clathrin baskets from the in situ samples indicate their localization in presynapse I), nonsynaptic neurite compartment II), and postsynapse III). Clathrin baskets are marked as yellow circles. (*B*) Distances to the cell membrane from clathrin baskets, CCVs, and SVs in on-grid-grown neurons. (*C*) Clathrin baskets as seen in cryo-ET volumes from on-grid-grown neurons and their StA. Clathrin cages are shown with yellow curves. (*D*) Isolated clathrin baskets and their StA. (*E*) Volume-rendered representations of clathrin cages shown to scale for isolated and in situ CCVs, endosomes, and empty clathrin baskets. (Scale bars *A*, *C*, *D*: 50 nm.) (*F*) A scheme for the cycle of SVs turnover highlighting the role of clathrin in the process.

We observed clathrin baskets without vesicles inside both in situ and in the isolated vesicle preparations (Fig. 4*A* and *SI Appendix*, Fig. S8 *A, B* and *E*). In neurons, empty cages were observed at presynapses (14%), postsynapses (52%), and extrasynaptic (34%) compartments (Fig. 4*A*). We further calculated and compared individual particle-to-membrane distances for empty clathrin baskets, CCVs, and SVs in on-grid-grown cells (Fig. 4*B*). The distribution suggests that the empty clathrin baskets (mean ~190 nm) and CCVs (mean ~150 nm) tend to be positioned closer to the membranes than SVs (mean ~228 nm; *SI Appendix*, *SI Materials and Methods* and Dataset S6). The respective pairwise distributions comparison of empty baskets versus SVs and CCVs versus SVs shows their statistical difference (*P* = 2.2e-4 << 0.05 and *P* = 3.2e-8 << 0.05, respectively), while for empty baskets versus CCVs it does not (*P* = 0.95 > 0.05; *SI Appendix*, *SI Materials and Methods* and Dataset S6).

The average radius of 149 empty clathrin cages from mouse brain tissue was ~36 nm, which is significantly smaller than clathrin cages with a vesicle inside (Fig. 4 *C*–*E*, and *SI Appendix*, Fig. S8 *C*). Ninety-two empty clathrin cages from on-grid-grown neurons had radii of 30-38 nm. We performed StA for empty clathrin cages in both datasets (in situ and isolated vesicles), and determined low-resolution structures (Fig. 4 *C* and *D* and *SI Appendix*, Fig. S3, S8*D*), showing disordered density inside the cages (*SI Appendix*, Fig. S8*D*). The structure obtained from the brain preparation contained a hexamer interacting with four pentamers and one hexamer, and one unclearly resolved polygon, potentially C2-basket geometry (15) (marked in *SI Appendix*, Fig. S8*D*).

Furthermore, we observed large ellipsoidal vacuoles partially coated with clathrin, resembling endosomes, both in situ and in the isolated vesicle preparation (*SI Appendix*, Fig. S9). We performed StA of the membrane segments containing clathrin coats using the available number of particles (n = 27, *SI Appendix*, Fig. S3). The resulting structure had an icosahedral symmetry with 1 pentamer, 4 hexamers, and 1 unclear polygon (Fig. 4*E* and *SI Appendix*, Fig. S3). Thus, our structures of empty clathrin cages showed a different symmetry type compared to the clathrin coats containing a vesicle inside or located at endosomes.

## Discussion

In this study, we combined in situ and ex vivo cryo-ET with biophysical methods, and structural and statistical analysis to perform a structural and morphometric characterization of proteins associated with SVs and CCVs. SVs isolated from mouse brains and SVs within neurons cultured on EM gids showed a comparable overall morphology, mass spectrometry confirmed a high abundance of SV proteins in our isolated vesicle preparation. Due to the lower sample thickness, our ex vivo SV dataset provided a higher signal, which allowed us to characterize and classify proteins integrated into the SV membrane and located inside the SV lumen. Structural analysis of these proteins involving StA has not been possible because of their heterogeneity and small size: VAMP2—18 kDa (43), Syp—38 kDa (44), synaptotagmin-1—65 kDa (45). A larger size would be required for structural analysis: recently, a structure of a 120 kDa mostly membrane-embedded protein was reported at an intermediate resolution of 16 Å (46). The highly abundant SV proteins such as Syp and NT transporters are membrane-embedded, which further limits their detection by cryo-ET, creating the observed apparent empty membrane. Nevertheless, we were able to classify observed protein densities into four categories and matched them with AlphaFold2 predictions of known SV proteins. Beyond that, we identified long IV protein densities. Since none of the SV proteins previously identified by mass spectrometry or fluorescence microscopy (1, 3, 5, 7) fitted into these densities, we suggest that extracellular adhesion proteins like cadherins account, at least in parts, for these densities. A possible explanation for their IV localization is that adhesion molecules are internalized during endocytosis, which is supported by our finding of long protrusions also inside CCVs. SVs may transport adhesion molecules toward the synapse, where they have been observed (33).

Even though intact SVs display a convoluted matrix with high protein density, we were able to characterize the abundance, distribution, and structure of V-ATPases. We showed that SVs had an average of 1.4 V-ATPases per SV, randomly distributed on their surface, suggesting that the positioning of V-ATPases is not crucial for their vesicular function. We produced an intermediate-resolution structure of the V-ATPase by StA and observed an ordered density next to its transmembrane domain, potentially forming a functional complex. We performed an integrative structural biological analysis of the candidate proteins and attribute the observed density containing a transmembrane segment and a small IV domain to the protein Syp for the following reasons: i) fitting of several candidate proteins to the density yielded the best results for Syp, synaptoporin, and Syp-like protein 1; ii) our proteinase K assay indicated that Syp is highly protected, supporting its mostly membrane-embedded and lumenal localization (*SI Appendix*, Fig. S2 *C* and *D*); iii) our LuTHy assay showed an interaction between human Syp and the V-ATPase Voe1 subunit. Furthermore, previous cross-linking MS data indicated direct interactions between Syp and V-ATPase and between VAMP2 and both Voa1 and Syp (5). Thus, it is likely that VAMP2, Syp, and the V-ATPase Vo region form complexes, as already suggested before (36). Although it was not possible to position VAMP2 in our structure due to its small size of 18 kDa, a single transmembrane helix, and a potentially less stable interaction, our LuTHy assay corroborated the presence of such an interaction, as it demonstrated strong pairwise interactions between VAMP2 and Voe1 and Syp. Independent from our study, two recent high-resolution structures of the rat and mouse V-ATPase showed that the V-ATPase forms a complex with Syp (39, 40). In Syp^-/-^ mice, the extra density, corresponding to Syp, was missing (40). These reports revealed high-resolution structures of the Syp–V-ATPase complex by single-particle cryo-EM on a pure SV fraction (39, 40). In our study, we complemented the moderate-resolution subtomogram average from cryo-ET with integrative structural biology analysis, leading to the same protein complex assignment.

The interactions between V-ATPase, Syp, and VAMP2 likely constitute a control mechanism to generate and maintain fully functional SVs over the rounds of SV recycling (47). The loss of the SNARE protein VAMP2 leads to perinatal lethality in homozygous knockout mice, likely because synaptic exocytosis is almost completely blocked (48). Likewise, acute photoinactivation of V-ATPase subunits was shown to strongly impair NT release (49). In contrast, Syp^-/-^ mice have a normal life expectancy and only show mild behavioral alterations (48) as well as a higher susceptibility for kainic acid-induced seizures (40). Syp^-/-^ mice, as well as humans harboring mutations in the Syp gene, display learning deficits and intellectual disabilities (50, 51). While Syp is not required for baseline neurotransmission (51), a stronger short-term depression has been observed during sustained synaptic transmission in the absence of Syp (52). This observation has been explained by the impaired Syp-dependent retrieval of VAMP2 during endocytosis and SV reformation (51, 52). Yet, the reduced VAMP2 copy number observed in Syp^-/-^ mice still exceeds the minimum number of VAMP2 copies necessary for evoked NT release (53). Beyond that, neither our analysis nor one of the recently published high-resolution cryo-EM studies of V-ATPases (39, 40) indicated a stable and ordered interaction between the V-ATPase and VAMP2. Likewise, our AlphaFold3-based structural prediction could not reliably position VAMP2, suggesting that the interactions of V-ATPase and VAMP2, as well as Syp and VAMP2, are more flexible.

Taking into account that Syp^-/-^ mice had almost doubled copy numbers of V-ATPases per SV (40), it is likely that the Syp–V-ATPase interaction safeguards the formation of functional SVs with an optimal number of V-ATPases required for NT loading. Whether a higher abundance of V-ATPases, as seen in the Syp^-/-^ mice, has functional consequences for synaptic transmission and behavior, needs to be studied further. In this regard, more V-ATPases per SV could lead to problems with NT loading and an imbalance between excitatory and inhibitory synaptic responses, typically associated with a higher susceptibility for seizures like in the Syp^-/-^ mice (40)

While the Vo region of the V-ATPase remains membrane-embedded during exocytosis and SV recycling, the V1 region is disassembled shortly before fusion and needs to be reassembled afterward. Previous studies indicated that the V1 domain proteins might be detected at the plasma membrane and at CCVs (18, 20). With our cryo-ET analysis of ex vivo CCVs, we now confirmed that the Vo and V1 may assemble at CCVs, indicating that the V1 region may be recruited already at an early stage of SV reformation. Our investigation of CCVs further revealed that not all clathrin coats were fully assembled.

We identified partially uncoated CCVs both in situ and in brain preparations, where adding ATP led to a higher abundance of partially coated CCVs. Partially assembled clathrin coats at synapses were previously described using classical transmission electron microscopy on thin slices through chemically fixed and stained samples (54). In our manuscript, we observed partially assembled clathrin coats in 3D using cryo-ET in natively preserved samples, without potential artifacts of fixatives, dehydration, and heavy metal staining. Notably, clathrin coats in most of the observed CCVs had an icosahedral symmetry. We propose that these vesicles initially arise from the fission of clathrin-coated pits, but subsequently undergo stepwise uncoating, which typically involves the proteins synaptojanin, Hsc70, and auxilin and is ATP-dependent (55, 56). We observed most CCVs in situ in post- and extrasynaptic neuronal compartments. Taking into account that the presynapse is filled with SVs, of which a fraction constantly undergoes exocytosis and clathrin-dependent SV reformation (55, 57), it is surprising that the fraction of CCVs is comparatively low, especially in relation to the high number of SVs. This observation underlines the optimized and tightly regulated coupling of exo- and endocytosis, which is required particularly during prolonged synaptic activity (58). In contrast, activity-independent CME taking place at endocytic zones farther away from the presynapse is slower and only promoted when necessary (59). Thus, we were able to capture the intermediate stages of the uncoating process enriched close to these endocytic zones. Since we did not detect any differences in the size, organization, or symmetry of CCVs from presynapses versus post- or extrasynaptic regions, we expect that the individual steps of CME are conserved between different neuronal compartments and different cellular functions.

The role of empty clathrin baskets in synapses, as observed by us and other independent studies (60, 61), remains unclear. Studies have demonstrated that clathrin can be disassembled and reassembled into cages of different symmetry in vitro (62) and in cells (60, 63). The empty baskets may thus arise from clathrin polymers preassembled without a vesicle inside, which was suggested earlier based on fluorescent microscopy experiments (64). These preassembled baskets could display a clathrin reservoir close to endocytic zones to facilitate the fast recruitment of clathrin triskelia or even partial lattices. Alternatively, the empty clathrin baskets may result from incomplete vesicle uncoating: since the vesicle uncoating initiates at hexagon corners (15), the relative abundance of pentamers increases over time. This may facilitate the formation of C2-symmetry baskets. Whether these baskets display a reservoir for faster clathrin recruitment and assembly or whether they are deposited for subsequent degradation, remains to be examined further.

In conclusion, our analysis provided insights into the molecular architecture of SV and the mechanisms of their assembly and recycling. Further progress in understanding the molecular landscapes of individual SVs may be expected from the ongoing developments in cryo-ET hardware, such as phase plates (65) and improvements in detectors and software. Furthermore, combinations of cryo-ET with labeling techniques and complementary information about PPI may provide further insights into the molecular architecture of SVs, their functional parts, and associated diseases.

### Animals.

All experimental procedures involving the use of mice were carried out in accordance with national and institutional guidelines. C57/BL6N wildtype mice for SV preparations in the study were bred at the Leibniz Research Institute for Molecular Pharmacology. Hippocampal primary culture neurons were prepared from P0-P2 C57/BL6N wildtype mice provided by Charité-Universitätsmedizin Berlin.

## Methods

### Culture of Primary Hippocampal Neurons.

Preparations of astrocytes and neurons from P0-P2 mice were performed as described previously (66). A feeder layer of astrocytes isolated from mouse cortices of either sex was seeded on collagen/ poly-D-lysine coated 6-well plates and cultured for 1 to 2 wk at 37 °C in DMEM containing 10% fetal calf serum (FCS) and 1% penicillin/streptomycin. Glia proliferation was arrested by the addition of 5-fluoro-2-deoxyuridine (8 μM) and uridine (20 μM) overnight and the medium was subsequently replaced by Neurobasal-A medium supplemented with B-27, Glutamax, and penicillin/streptomycin prior neuron seeding. Quantifoil R3.5/1 AU holey carbon 400 mesh grids were glow-discharged, coated with collagen/poly-D-lysine, and placed on pedestals on top of 1 to 2 wk old astrocytes. Primary hippocampal neurons were plated with a density of 150,00 to 200,000 cells/well and grown for 17 d at 37 °C until further use.

### Synaptic Vesicle Enrichment From Mouse Brain Tissue.

SV from murine neurons were isolated as described previously (1, 67, 68), stopping after the LP2 fractionation. All steps were performed at 4 °C. Briefly, the hippocampus, cortex, and cerebellum of six 40-d-old mice were extracted and homogenized in homogenization buffer (HB; 4 mM HEPES pH 7.4, 320 mM sucrose, 1 mM PMSF, protease inhibitor mix) with nine strokes in a Dounce homogenizer at 900 rpm. Tissue debris was removed by centrifugation at 800×*g* for 10 min. The supernatant was centrifuged at 10,000×*g* for 15 min and the pellet containing synaptosomes was collected and washed with HB. Synaptosomes were swelled in a hypotonic buffer (1:10 HB in H_2_O) and lysed with three strokes in the Dounce homogenizer at 2,000 rpm. Isotonic conditions were restored and the mixture was centrifuged at 25,000×*g* for 20 min. Subsequently, the supernatant was centrifuged at 200,000×*g* for 2 h. The pellet was resuspended in 150 µL HB and the protein concentration was determined by Bradford assay and negative staining. As we aimed for a faster purification protocol to maximally preserve the molecular architecture of SVs, we skipped the affinity or GF columns, adopted in other protocols, such as Wang and Coupland (39, 40).

### Sample Preparation for Cryo-ET.

Four biologically replicated sample preparations, 20 μl of the vesicles sample (c = 0.15 mg/mL) were mixed with five- or ten-nanometer gold fiducial markers (purchased from the Cell Microscopy Core, Utrecht University) with the ratio of 1:10. Four μL of this mixture was deposited to glow-discharged cryo-EM grids (UltrAuFoil R1.2/1.3, Quantifoil Cu 2/2, or Quantifoil AU 100 Holey carbon Films R2/1), blotted for 3 s at relative humidity 98% and temperature 4 °C and plunge-frozen into liquid ethane (Vitrobot).

Three types of samples were prepared — one without additional factors, the second one with V-ATPase inhibitor bafilomycin A1 to prevent proton translocation through V-ATPase, and the third one — with ATP. The ATP-containing cryosample was prepared as described before (69). Briefly, 3 μL of freshly isolated SVs were mixed with 1 μL of ATP solution in PBS containing MgCl2 (final concentration of ATP c = 4.5 μM, final concentration of MgCl_2_ c = 5 μM), and incubated on ice for 5 min before applying on the grid. The bafilomycin A1-containing cryosample was prepared as described previously (70). A separate aliquot of SV was incubated with bafilomycin A1 (final c = 90 nM) on ice for 15 min prior to freezing. The initial approach was to perform subtomogram classification to obtain several conformational states of V-ATPase in the native environment. However, the achieved resolution for each of the three datasets (~25 Å) did not allow us to detect differences. Therefore, all datasets were processed together, aiming at separating V-ATPase particles from false positive particles, followed by the analysis of V-ATPase distribution over vesicles and potential clustering.

For the neurons grown directly on grids, a freezing solution was prepared containing 140 mM NaCl, 2.4 mM KCl, 10 mM HEPES, 10 mM glucose, 4 mM CaCl_2_, 1 mM MgCl_2_, 3 µM NBQX, 30 µM bicuculline, TTX (control solution only), (~300 mOsm; pH=7.4). 10 nm BSA-gold in freezing solution (OD~2) was used as fiducial markers. Grids were briefly washed in freezing solution prewarmed to 37 °C, then transferred to the plunge freezer (Vitrobot). 4 µL fiducial marker-containing freezing solution was added to the grid prior to blotting for 16 s (backside blotting, blot force 10) at 37 °C and 80% relative humidity and plunge-freezing into liquid ethane.

Cellular tomograms used in this study were originally prepared for a study in which two single action potentials were induced via optogenetic stimulation short before sample vitrification. For this purpose, the samples were infected with a lentivirus for the expression of the channelrhodopsin-2 variant ChR2(E123T/T159C) (71, 72). The first action potential was induced approximately 100 ms, the second one 2 to 5 ms before freezing. NBQX and bicuculline were added to the freezing solution (total incubation time < 2 min) to avoid network activity. Previous studies showed that clathrin-dependent SV reformation takes place seconds after membrane internalization, even during ultrafast endocytosis (73). This means that the two induced single action potentials did not have any impact on the findings of our study, particularly when taking into account that neurons in mass culture show a high rate of spontaneous network activity (57). Channelrhodopsins as tools for optogenetic stimulation are commonly accepted and used in the field of neuroscience because optogenetic stimulation is supposed to mimic physiological neuronal activity better than other methods like chemical or electrical stimulation without disturbing neuronal morphology and function. We carefully compared optogenetically stimulated and unstimulated control data prepared in parallel and could not find differences between the two datasets. Therefore, we pooled these two samples and referred to them as “in situ” data.

### Cryo-ET Data Collection, Image Processing, and Subtomogram Averaging.

Three dose-symmetrical and four dose-asymmetrical (74) data collection sessions were performed on a TFS Titan Krios electron microscope operated at 300 kV with a Gatan Quantum energy filter with a slit width of 20 eV and a K3 (Gatan) direct detector operated in counting mode. For several data collection, different total exposures were used: ~128 e^−^/Å^2^, ~270 e^−^/Å^2^, ~229 e^−^/Å^2^, ~271 e^−^/Å^2^, ~213 e^−^/Å^2^ was equally (for 3 datasets) (75), or unequally (for 4 datasets) (74) distributed between 31, 35, or 37 tilts. Ten frame movies were acquired for each tilt. The details of data collection are given in SI *Appendix*, Table S1. The number of collected tomograms: hippocampal neurons - 88 tilt series were collected (superresolution pixel size = 3.07 Å) using PACE-tomo (76); for isolated SV fractions - 719 tilt series (superresolution pixel size = 0.84 Å), 438 of which were collected with PACE-tomo (76).

Data processing was streamlined using TomoBEAR (77). The aligned frames were motion-corrected using MotionCor2 (78). Tilt-series alignment was performed by DynamoTSA (79) and manually inspected and refined in IMOD (80, 81), using the 10-nm or 5-nm gold fiducial markers. For each projection, the defocus values were measured by Gctf (82), and CTF correction was performed using ctfphaseflip (82) from IMOD. Weighted backprojection in IMOD was used to produce sixteen times binned reconstructions of 719 tomograms generated from CTF-corrected, aligned stacks. Particles were picked manually (V-type ATPases and clathrin baskets) from 16-times binned superresolution tomograms collected from the brain tissue (voxel size: 13.44 Å), with further extraction of 60×60×60 sized boxes (V-ATPase) and 116×116×116 sized boxes (empty clathrin baskets). Subtomogram averaging was performed in Dynamo (79) and Relion 4 (83).

Further details can be found in *SI Appendix*.

## Supplementary Material

Appendix 01 (PDF)

Dataset S01 (XLSX)

Dataset S02 (XLSX)

Dataset S03 (XLSX)

Dataset S04 (XLSX)

Dataset S05 (XLSX)

Dataset S06 (XLSX)

Dataset S07 (XLSX)

## Data Availability

The mass spectrometry proteomics data have been deposited to the ProteomeXchange Consortium via the PRIDE partner repository with the dataset identifier PXD045356 (84). Subtomogram average structures were deposited to the EMDB with Accession No: EMD-18556 (V-ATPase) (85), EMD-18557 (V-ATPase transmembrane partner) (86), EMD-18572 (a segment of CCV isolated from mouse brain tissue) (87), EMD-18574 (a segment of a clathrin-covered endosome isolated from mouse brain tissue) (88), EMD-18578 (empty clathrin basket from mouse brain) (89), EMD-18584 (clathrin triskelion from primary hippocampal neuron culture) (90), EMD-18568 (clathrin triskelion from mouse brain tissue) (91), EMD-18582 (empty clathrin basket from primary hippocampal neuron culture) (92), EMD-18583 (a segment of a CCV from primary hippocampal neuron culture) (93).
