# Supplementary material for: Molecular architecture of synaptic vesicles
Source: Proc Natl Acad Sci U S A. 2024 Nov 27;121(49):e2407375121. doi: 10.1073/pnas.2407375121 (PMC11626200; doi:10.1073/pnas.2407375121)
Supplement: Supplementary file 1 — Appendix 01 (PDF) [file pnas.2407375121.sapp.pdf]

Supporting Information for

Molecular architecture of synaptic vesicles

Uljana Kravčenko[a,b], Max Ruwolt[c,1], Jana Kroll[d,e,f,1], Artsemi Yushkevich[a,g,1], Martina Zenkner[h,1], Julia Ruta[a,c,e], Rowaa Lotfy[a,i], Erich E. Wanker[h], Christian Rosenmund[f], Fan Liu[c,j], Mikhail Kudryashev[a,k,2]

a. In situ Structural Biology, Max Delbrück Center for Molecular Medicine in the Helmholtz Association (MDC), Berlin, Germany.

b. Department of Biology, Humboldt University of Berlin, Germany.

c. Leibniz Research Institute for Molecular Pharmacology, Berlin, Germany.

d. Structural Biology of Membrane-Associated Processes, Max Delbrück Center for Molecular Medicine in the Helmholtz Association (MDC), Berlin, Germany.

e. Institute of Chemistry and Biochemistry, Freie Universität Berlin, Germany.

f. Institute of Neurophysiology, Charité-Universitätsmedizin Berlin, Germany.

g. Department of Physics, Humboldt University of Berlin, Germany.

h. Neuroproteomics, Max Delbrück Center for Molecular Medicine in the Helmholtz Association (MDC), Berlin, Germany.

i. Institute of Pharmacy, Freie Universität Berlin, Germany.

j. Charité-Universitätsmedizin Berlin, Germany.

k. Institute of Medical Physics and Biophysics, Charité-Universitätsmedizin Berlin, Germany.

1. Indicates equal contribution.

2.Correspondence to: M.K. (mikhail.kudryashev@mdc-berlin.de);

This PDF file includes:

Figures S1 to S9

Table S1 to S5

SI Materials and Methods

References for Supplementary Material

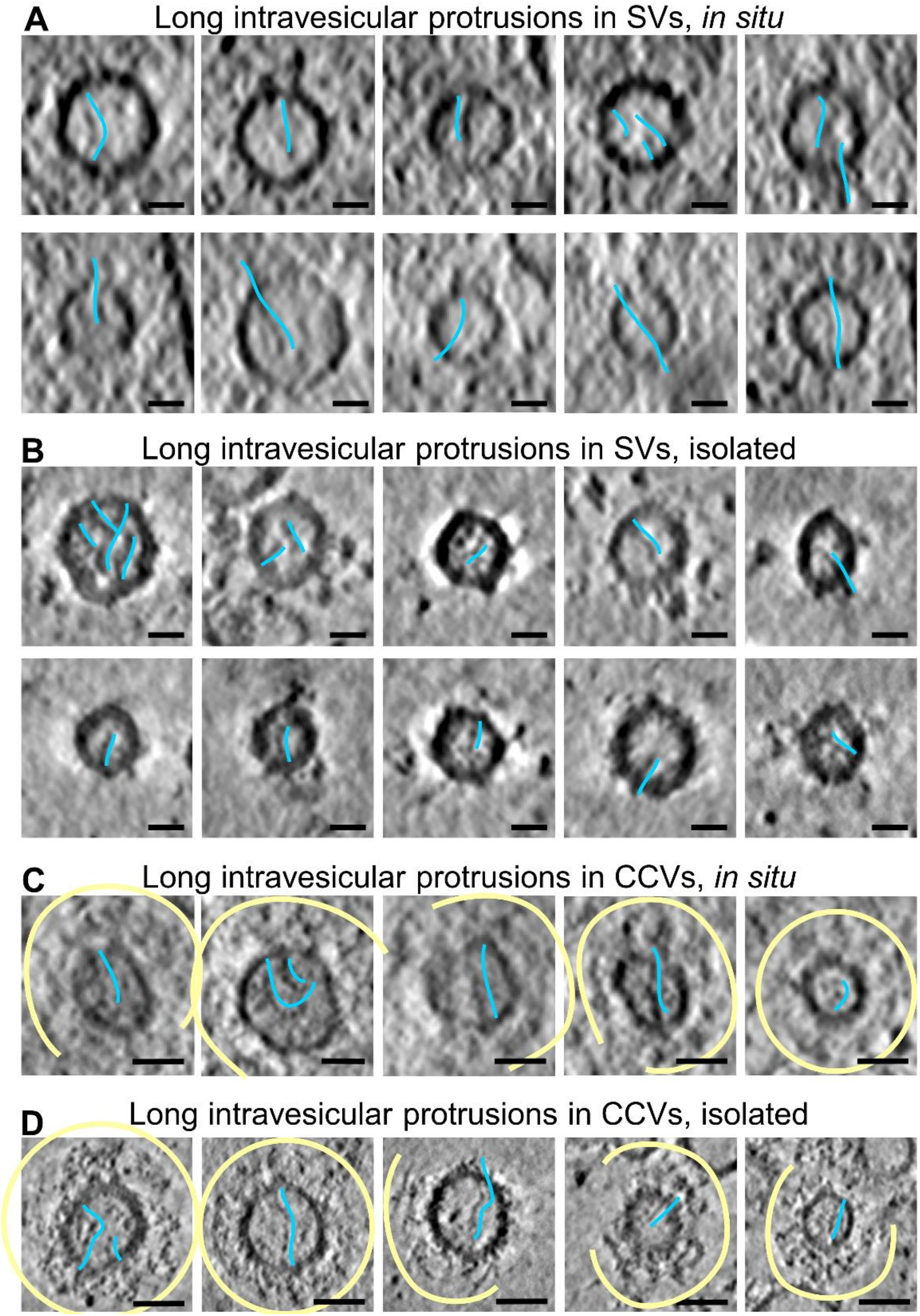

Fig. S1. Examples of long intravesicular densities in SVs and CCVs. Slices through tomograms of (A) *in situ* SVs (primary hippocampal neurons) (B) isolated SVs (C) *in situ* CCVs (primary hippocampal neurons) (D) isolated CCVs showing long inner protrusions (marked with blue curves next to the protrusion densities). Scale bars (A-B): 25 nm, (C-D): 50 nm. Clathrin cages are shown in yellow in (C, D).

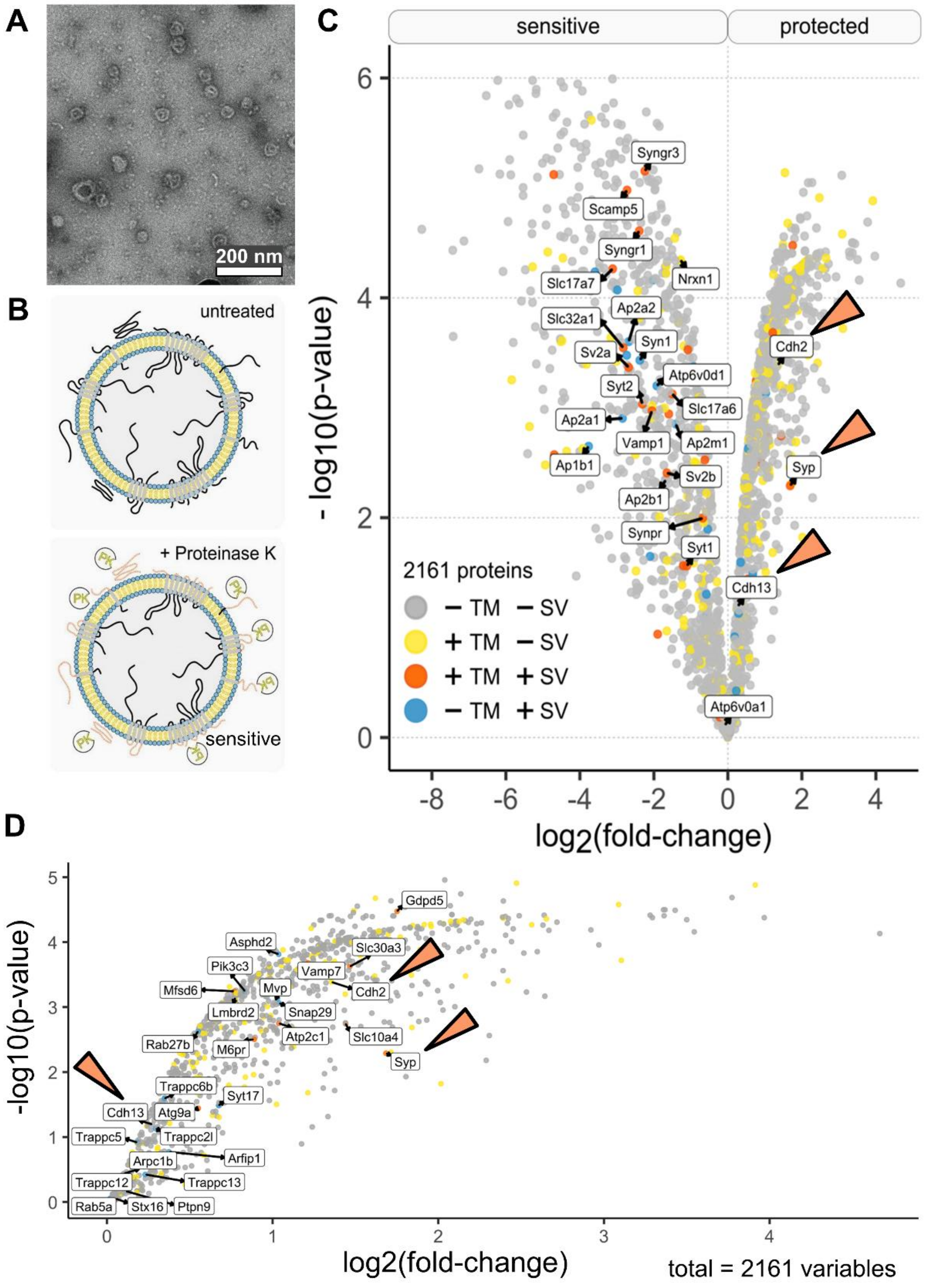

Fig. S2. Mass spectrometry and PK-analysis on SVs. A) Negative staining on the isolated SV sample, scale bar: 200 nm. B) Schematic representation of enzymatic hydrolysis of surface accessible protein domains on isolated murine SVs upon PK treatment. C) Label-free quantitative analysis of PK-treated SVs. Volcano plot with log2 fold-change and -log10 p-value of 2161 proteins from 3 replicates shows proteins susceptible to (negative fold-change) and protected from (positive fold-change) PK treatment. Known protein localization within synaptic vesicles (+SV) and DeepTMHMM-predicted transmembrane domains (+TM) are highlighted. PK-protected proteins are pointed with orange arrows. D) An extended scatter plot represents all PK-protected proteins.

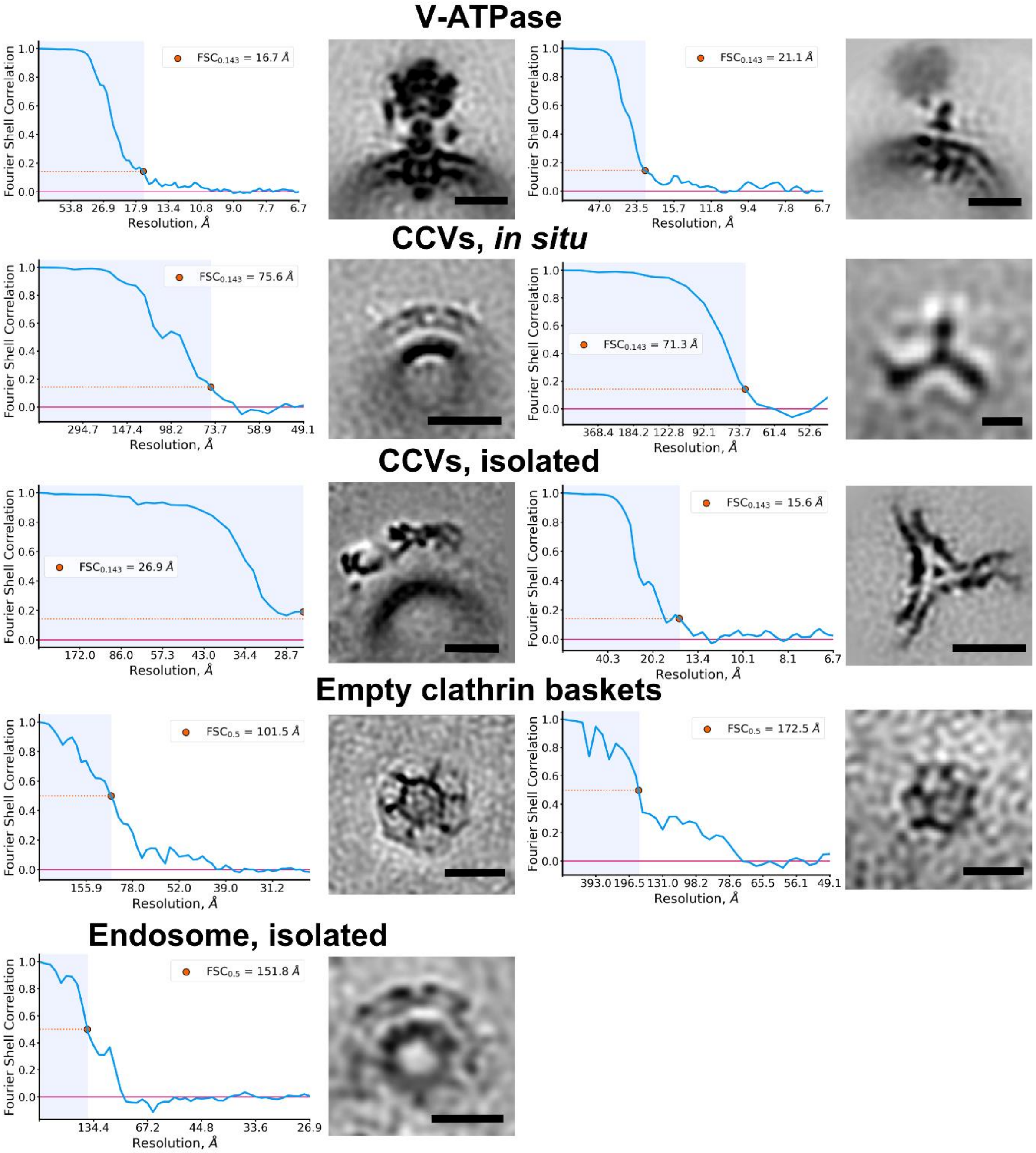

Fig. S3. Structures and resolution estimates of the molecules from the main text. Fourier Shell Correlations (FSC) curves for the in-text described StA structures. For independent half-set refinements - a low threshold of FSC=0.143 is set up. For empty clathrin baskets and an endosome due to the low number of particles, we could only perform refinement of non-independent half-sets and used a higher threshold of FSC=0.5 for resolution measurement. Scale bars: (V-ATPase) both - 10 nm; (CCVs, *in situ*) left - 50 nm, right - 15 nm; (CCVs, isolated) left - 25 nm, right - 15 nm; (Empty clathrin baskets) both - 50 nm; (Endosome) 50 nm.

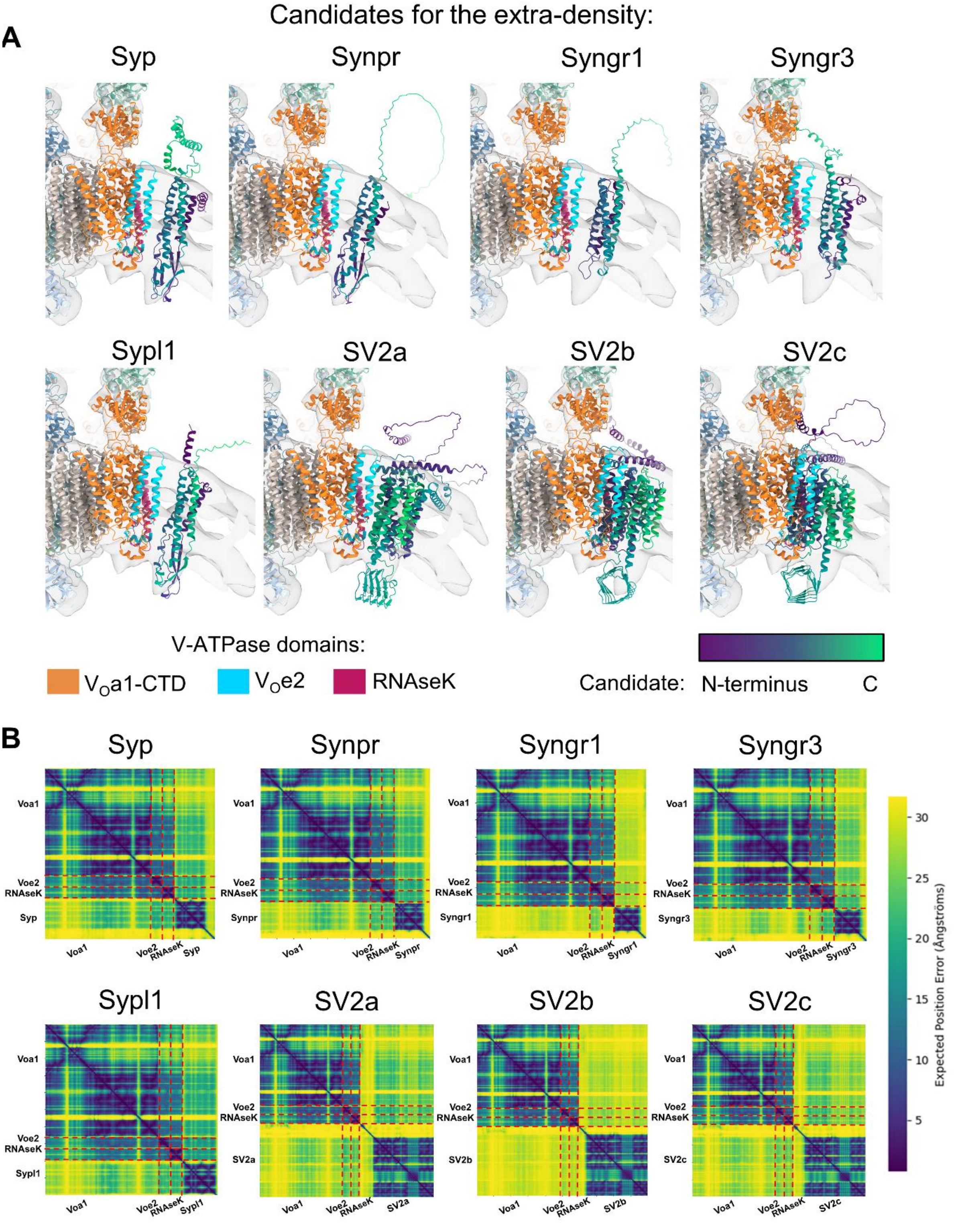

Fig. S4. Candidates for a protein density found in proximity to V-ATPase. A) AlphaFold3[1]-predicted candidate models fit to the resolved density next to the V-ATPase Vo domain. V-ATPase model: 6wm2[2] (PDB). Candidates are colored in a purple-to-yellow gradient, where purple corresponds to the N terminus and green - to the C-terminus. B) Predicted Aligned Error (PAE) plots - an AlphaFold3 prediction metrics, describing the residue-to-residue expected positional error. The distance between each candidate and the V0 proteins is within ~20-30 Å of the residue positional error.

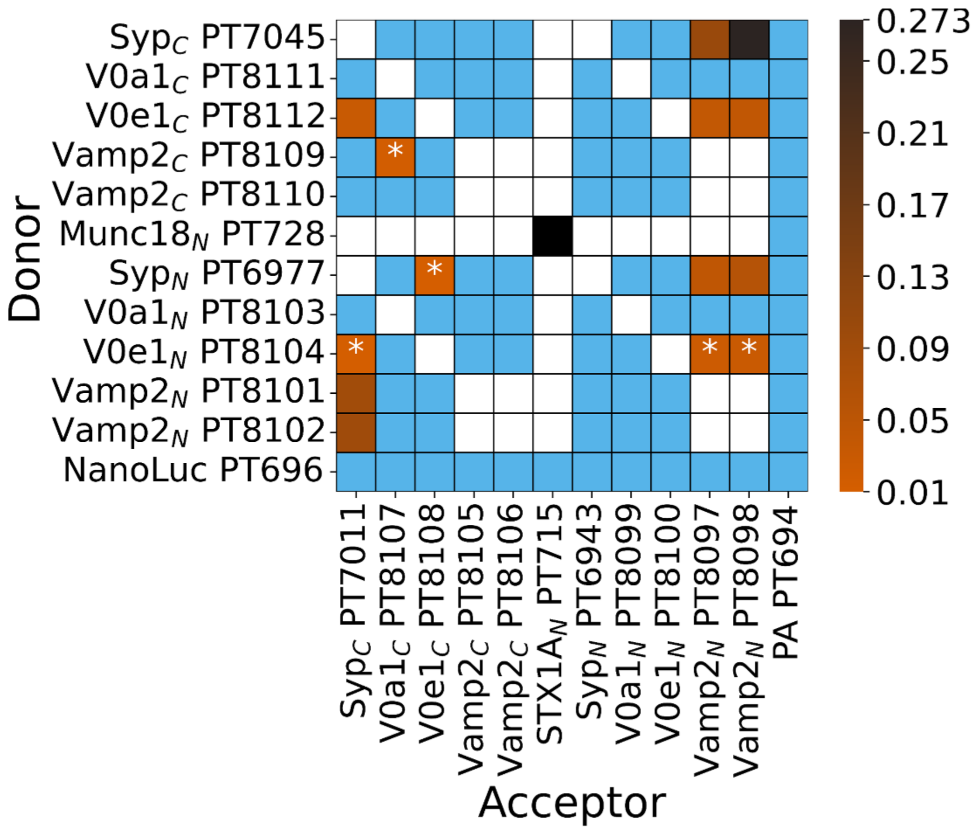

Fig. S5. LuTHy assay performed on V-ATPase Voe1, V-ATPase Voa1 domains, Syp and VAMP2. Control-corrected BRET (cBRET) ratios for the tested pairs of interacting partners as the heatmap, representing protein interaction strength with a color gradient from orange to black. The interactions with observed cBRET values below the cBRET cutoff of 0.01 (cBRET cutoff for cytoplasmic/nuclear/membrane proteins) are represented with blue color. The asterisks (*) annotations represent interactions with cBRET values ≥0.01 (the cutoff for cytoplasmic/nuclear/membrane proteins[3]) but <0.03 (the cutoff for membrane proteins only). The strength of a positive control pair (Munc18-N / STXN1A-N) is shown with a dark black color (cBRET = 0.546), while negative control pairs (NanoLuc-to-all, PA-to-all) are represented with blue color as well. Non-tested pairs are shown by non-colored/white cells.

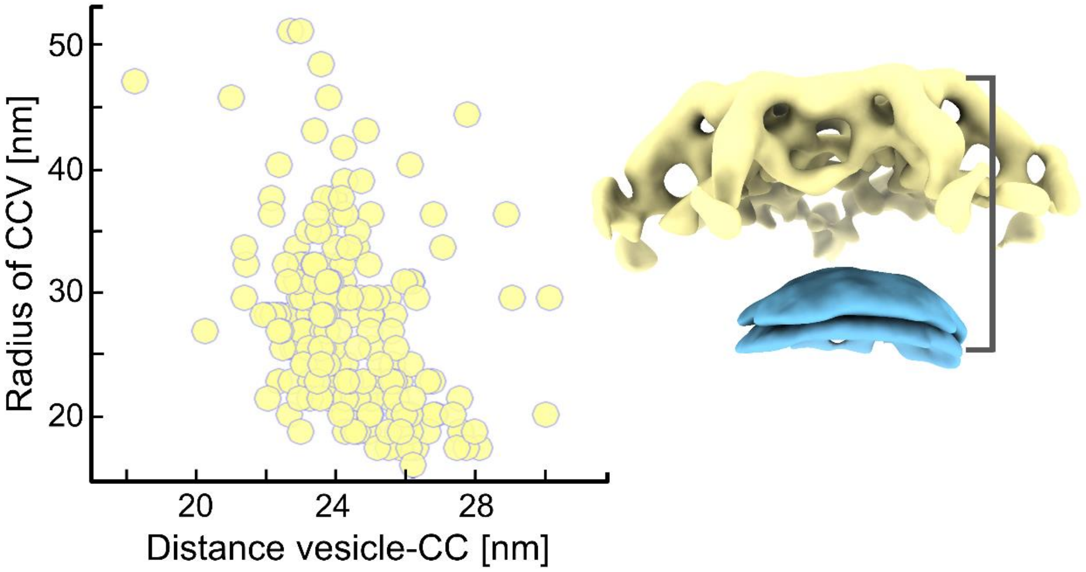

Fig. S6. CC-to-coated vesicle distance. Left: Scatter plot of CCVs radii (CC-enclosed vesicle center-to-membrane distances) and the vesicle membrane-to-CC distances. Right: A volume rendering (ChimeraX) of a CCV surface region, showing CC facet (top, yellow) and CC-enclosed vesicle surface (bottom, blue). The annotation line represents the distance definition for the vesicle-CC measurements.

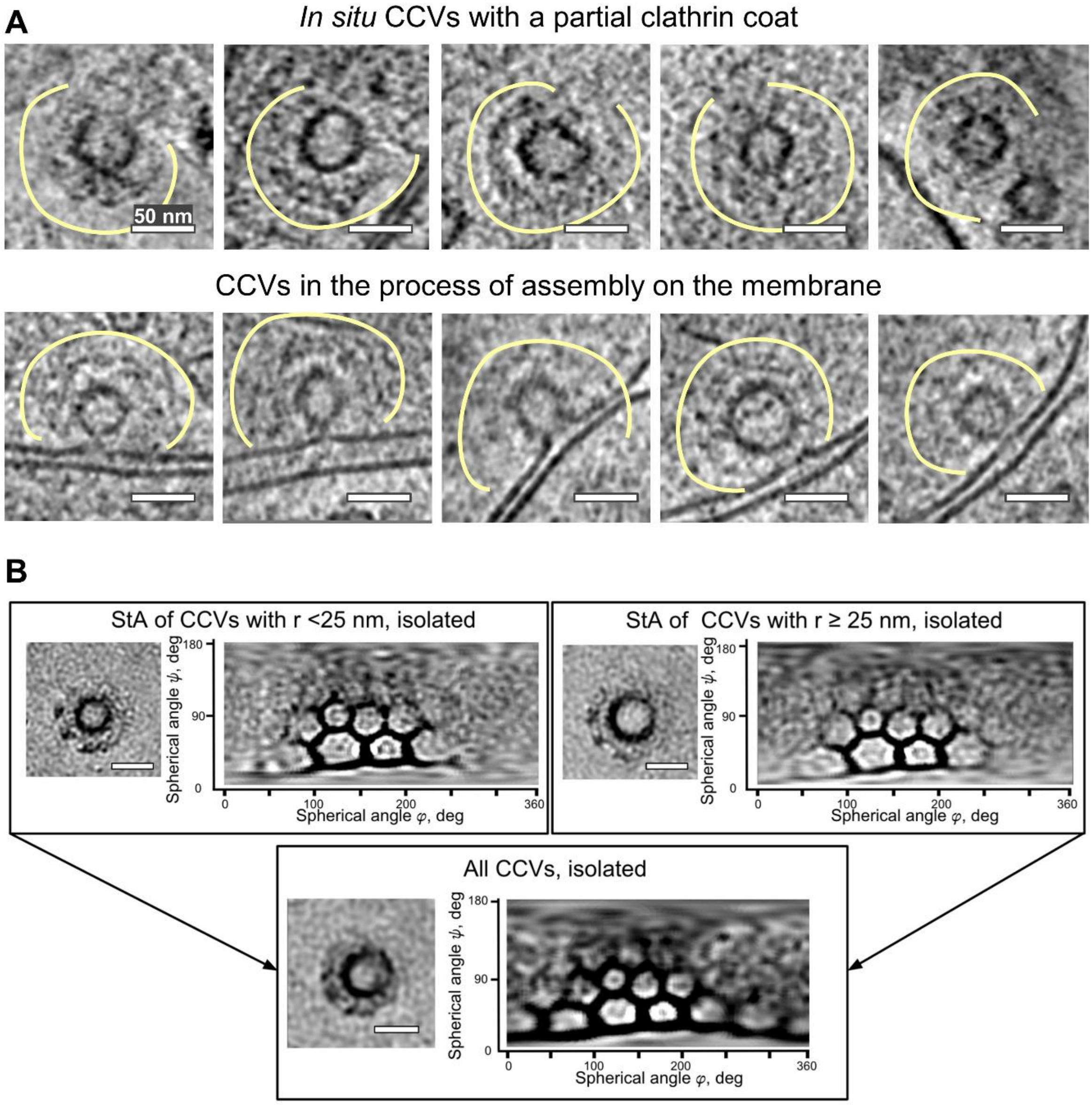

Fig. S7. CCVs. A) Upper row: CCVs with different percentages of clathrin coverage from the non-stimulated dataset. Lower: CCVs in the process of assembly on the membrane. Scale bar 50 nm. B) StA of CCVs and their spherical “unwrapping” in spherical angles coordinates (φ,ψ) of the image on the left.

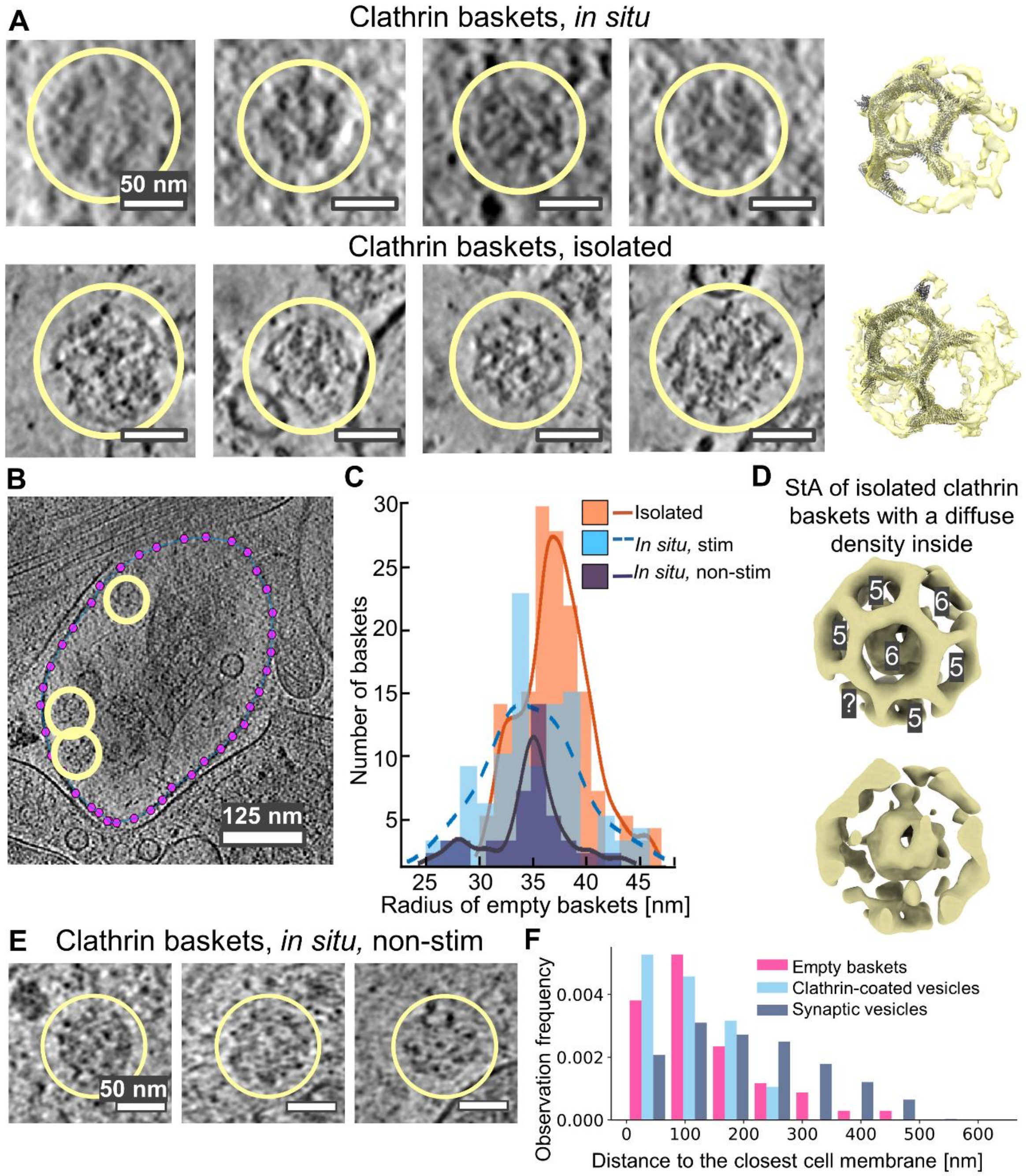

Fig. S8. Visualizations of non-vesicle-containing clathrin baskets. A) Slices through tomograms of single non-vesicle-containing clathrin baskets *in situ* (upper row) and in isolated fractions (lower row) and their StA structures with the fitted atomic models of the clathrin triskelions (PDB: 6SCT[4]) forming a pentagonal facet. The fitting is performed in ChimeraX. Scale bars: 50 nm. B) In-cell clathrin vesicle localization analysis: creating a membrane model using *Dynamo Catalogue*, scale bar: 125 nm. C) Distribution of non-vesicle-coating clathrin baskets radii in the isolated preparation (orange), in stimulated (blue) and non-stimulated (purple) cells. D) ChimeraX representation of an isolated basket StA without a vesicle inside. Pentagones or hexagons are shown with numbers 5 or 6 respectively. Two slices show a diffuse density inside. E) Examples of *in situ* clathrin baskets from non-stimulated neurons. Scale bar: 50 nm. F) Distribution of distances from CCVs and SVs to the closest cell membrane in non-stimulated neurons.

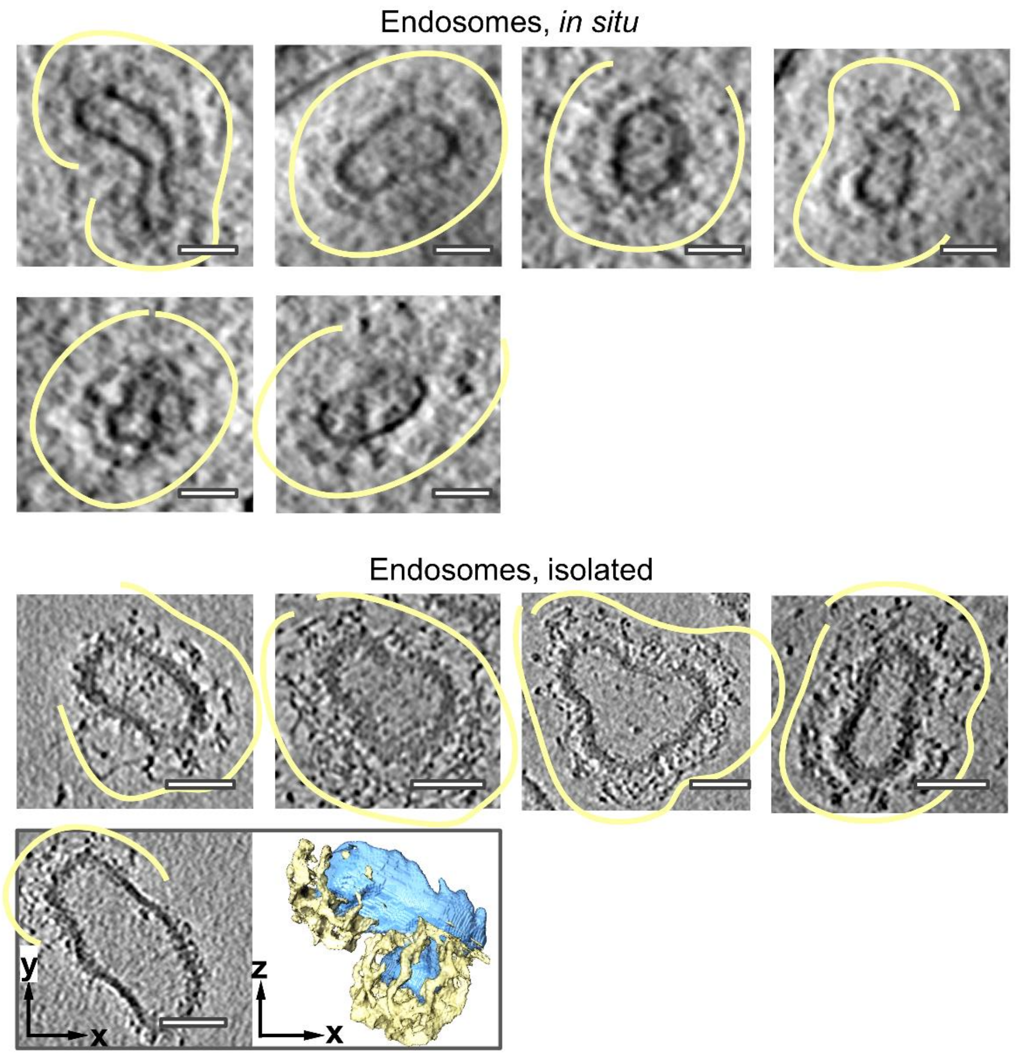

Fig. S9. Top: Endosomes, observed in neurons. Bottom: Endosomes, isolated from mouse brains, and the volume rendering of one of them using AMIRA (Thermo Fisher Scientific). Scale bars: 50 nm.

Table S1. Data collection and processing statistics for the StA.

	**V-ATPase**	**V-ATPase partner**	**CCVs, fragment, *in situ***	**Clathrin triskelion, *in situ***	**Clathrin baskets, *in situ***
Data collection and processing					
Microscope	Titan Krios G3i	Titan Krios G3i	Titan Krios G3i	Titan Krios G3i	Titan Krios G3i
Magnification	53,000 x	53,000 x	15,000 x	15,000 x	15,000 x
Voltage (kV)	300	300	300	300	300
Cs (mm)	2.7	2.7	2.7	2.7	2.7
Total electron dose ($e^-/Å^2$)	128…271	128…271	107	107	107
Defocus range (µm)	-3.5 to -5.5	-3.5 to -5.5	-3.5 to -6.0	-3.5 to -6.0	-3.5 to -6.0
Camera	Gatan K3	Gatan K3	Gatan K3	Gatan K3	Gatan K3
Pixel size (Å)	3.36	3.36	24.56	24.56	24.56
Number of tomograms	719	719	34	34	34
Symmetry imposed	C1	C1	C1	C1	C1
Initial number of particles	10204	10204	15789	9384	92
Final number of particles	5361	5361	1564	3658	34
Refinement method	Independent half-sets	Independent half-sets	Independent half-sets	Independent half-sets	Independent half-sets
Map resolution (Å)	16.7	21.1	75.6	71.3	172.5
FSC threshold	0.143	0.143	0.143	0.143	0.5

Table S1 (continue). Data collection and processing statistics for the StA.

	**CCVs, fragment, isolated**	**Clathrin triskelion, isolated**	**Clathrin baskets, isolated**	**Endosomes, fragment, isolated**
Data collection and processing				
Microscope	Titan Krios G3i	Titan Krios G3i	Titan Krios G3i	Titan Krios G3i
Magnification	53,000 x	53,000 x	53,000 x	53,000 x
Voltage (kV)	300	300	300	300
Cs (mm)	2.7	2.7	2.7	2.7
Total electron dose (e^-/Å^2)	128…271	128…271	128…271	128…271
Defocus range (µm)	-3.5 to -5.5	-3.5 to -5.5	-3.5 to -5.5	-3.5 to -5.5
Camera	Gatan K3	Gatan K3	Gatan K3	Gatan K3
Pixel size (Å)	13.44	3.36	13.44	13.44
Number of tomograms	147	147	147	5
Symmetry imposed	C1	C1	C1	C1
Initial number of particles	27498	9554	265	1073
Final number of particles	1702	8006	51	27
Refinement method	Independent half-sets	Independent half-sets	Independent half-sets	Independent half-sets
Map resolution (Å)	26.9	15.6	101.5	151.8
FSC threshold	0.143	0.143	0.5	0.5

Table S2. Candidates for the class 1 density - small extra-vesicular domains.

**Candidate class 1**	**Num per SV in reference[5]**	**Num of TM helixes**	**TM domain AA**	**Protein function**	**UniProt ID**	**Prediction**
Synaptotagmin -1	Synaptotagmins - 15.2	1	58-79	A component of Ca2+ sensor, which triggers the synchronous release of neurotransmitters in synapse[6].	P46096	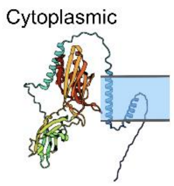
Synaptotagmin -2		1	61-87	Inositol-1,3,4,5-tetrakisphosphate (IP4) or inositol polyphosphate-binding protein, potentially involved in synaptic function[7].	P46097	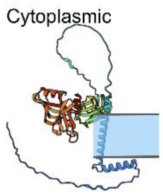
Synaptotagmin -3		1	55-75	Presynaptic Ca2+ sensor, which forces vesicle refilling and short-term synaptic plasticity[8].	O35681	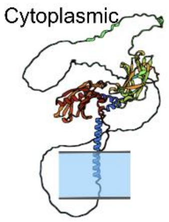
Rab3A	10.3	0	-	A GTP-binding protein, which is involved in synaptic vesicle transportation to the active zone and docking to the membrane[9].	P63011	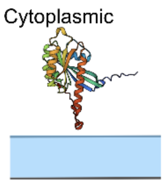

Synapsin-1	Synapsins - 8.3	0	-	Regulation of axonogenesis and synaptogenesis[10].	O88935	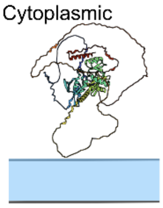
Synapsin-2		0	-	Desynchronizes γ-aminobutyric acid release in a Ca2+-dependent manner by interaction with presynaptic Ca2+ channels[11].	Q64332	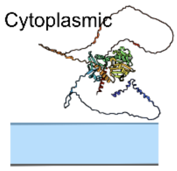
RalA	-	-	-	A GTP sensor for the GTP-dependent dense core vesicle exocytosis. Not essential for the general secretory pathways[12].	P63321	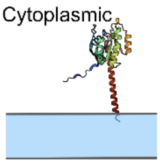
Vesicle-trafficking protein SEC22b	-	1	195-215	A component of SNARE proteins. Participates in vesicular transport between the ER and the Golgi apparatus[13].	O08547	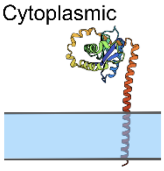
SCAMP1	0.8	4	156-176<br>182-202<br>219-239<br>262-282	Interacts with EH domain proteins and might participate in endocytosis by directing the assembly of clathrin coats at the	Q8K021	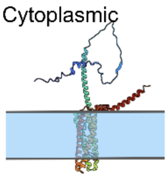

				plasma membrane[14].		
SCAMP3	-	4	169-189<br>200-220<br>236-256<br>277-297	Participates in the biogenesis of multivesicular endosomes. Important for EGF receptor sorting into multivesicular endosomes and its targeting to lysosomes by forming intraluminal vesicles within these endosomes *in vitro*[15].	O35609	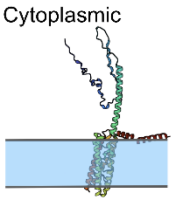

(*The N-terminus is shown in blue, and the C-terminus - in red)

Table S3. Candidates for the class 2 density - extra- and intra-vesicular domains.

Candidate class 2	Num per SV in reference[5]	Num of TM helixes	TM domain AA	Protein function	UniProt ID	Prediction
SV2a		12	170-190<br>206-226<br>234-254<br>263-283<br>295-315<br>335-355<br>448-468<br>599-619<br>627-647<br>652-672<br>686-708<br>713-731	Together with gangliosides, mediates the entry of Botulinum neurotoxin E into neurons[16].	Q9JIS5	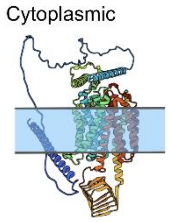
SV2b	SV2 - 1.7	12	111-131<br>149-169<br>183-203<br>206-226<br>238-258<br>278-298<br>391-411<br>536-556<br>566-586<br>593-613<br>627-649<br>654-672	Together with gangliosides, mediates the entry of Botulinum neurotoxin E into neurons[16].	Q8BG39	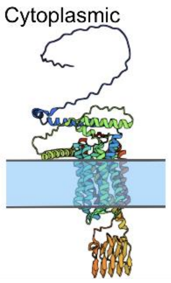
SV2c		12	155-175<br>192-212<br>227-247<br>249-269<br>281-301<br>321-341<br>438-458<br>579-599<br>610-630<br>637-657<br>671-693<br>698-716	Mediates a dopamine neuron function; its dysfunction leads to Parkinson's disease which may contribute to dopaminergic dysfunction[17].	Q69ZS6	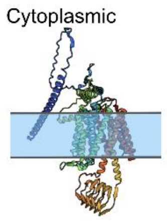

(*The N-terminus is shown in blue, and the C-terminus - in red)

Table S4. Candidates for the class 3 density - small intra-vesicular domain.

Candidate class 3	Num per SV in reference[5]	Num of TM helixes	TM domain AA	Protein function	UniProt ID	Prediction
Synaptophysin	31.5	4	26-49<br>108-131<br>139-162<br>201-224	Regulates endocytosis during and after neuron stimulation[18]. Regulates synaptic transmission *in vivo*. Redundant and essential in long and short synaptic plasticity. Not required for neurotransmitter release[19].	Q62277	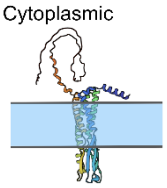
Synaptogyrin-1	2	4	24-44<br>72-92<br>104-124<br>149-169	Redundant and essential in long and short synaptic plasticity. Not required for neurotransmitter release[19].	O55100	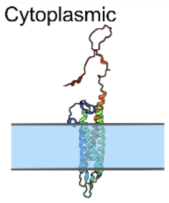
Synaptogyrin-3		4	30-50<br>70-90<br>105-125<br>148-168	Synaptogyrin-3 binding tau protein may cause synaptic dysfunction in progressive supranuclear palsy[20].	Q8R191	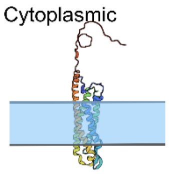
Synaptophysin-like protein 1	Not available	4	34-54<br>118-138<br>152-172<br>215-235	Under investigation.	O09117	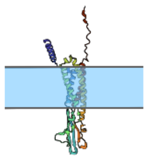

Synaptoporin		4	5-25<br>82-102<br>115-135<br>179-198	A putative channel protein of synaptic vesicles[21].	Q8BGN8	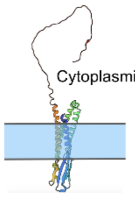

(*The N-terminus is shown in blue, and the C-terminus - in red)

Table S5. Candidates for the class 4 density-long intravesicular domain.

Candidate class 3	Num per SV in reference[5]	Num of TM helixes	TM domain AA	Protein function	UniProt ID	Prediction
Cadherin-13	NA	1	not annotated	Localized at inhibitory presynapses. CDH13 deficiency in iGABAs increases inhibition and thus decreases excitation/ inhibition balance[22].	Q9WTR5	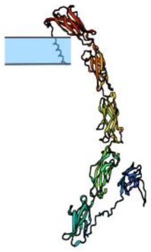
Cadherin-2	NA	1	725-745	Ca2+-dependent homotypic cell adhesion protein[23].	P15116	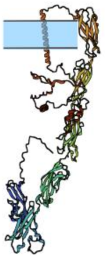

(*The N-terminus is shown in blue, and the C-terminus - in red)

SI Materials and Methods

Cryo-ET data processing and subtomogram averaging

For V-ATPase, three types of datasets were recorded - one without additional factors (450 tomograms), one with an inhibitor bafilomycin A (179 tomograms), and one with additional ATP (90 tomograms). First we manually identified, or "picked" 10204 V-ATPases on the surface of SVs. For structural analysis, 500 particles were used to perform the initial manual alignment in *dynamo_gallery*. The alignment parameters for the roughly aligned particles were further refined using Dynamo[24]. The particles were averaged to produce an initial average which was used as an initial reference to perform the global alignment of all subtomograms. Several rounds of multireference alignment and classification were performed in Dynamo, leading to the removal of suboptimal particles. Classification rounds were performed in several iterations, with the first range of 360° around the unit sphere. C1 symmetry was used and a wide soft-edged cylindrical mask. Aligned particles in good and bad classes were examined manually in *dynamo_gallery* to minimize the loss of good particles, which was important for further statistical analysis. In total, 5361 particles were identified as "good" ones and transferred to RELION-4.0[25] for further refinement and 3D classification, using 4-times binned super-resolution stacks and a protein-shaped mask created in ChimeraX[26]. The final 3D average originating from 5361 particles was refined to a resolution of 16.7 Å resolution. During processing, no symmetry was applied. We did not observe differences between the structures reconstructed from different datasets; the highest resolution structure was obtained by combining all three datasets. The same final particle set was used for further focused refinement on the Vo-a1 domain with the soft-edged cylindrical mask. This resulted in the 21.1 Å resolution map with a membrane protein density with a short intravesicular domain.

For empty clathrin baskets, both in cells and isolated ones, a similar Dynamo pipeline was used. The particles were picked manually. Sixteen-times binned super-resolution subtomograms were refined and 3D-classified using a spherical soft-edged mask in Dynamo. This resulted in low-resolution structures: 90 Å for the cage isolated from mouse brain tissue (51 baskets), and 160 Å for the cage from primary hippocampal neurons (34 baskets).

Sixteen-times binned super-resolution CCVs' subtomograms were picked using a spherical model in the *Dynamo Catalogue* system[27], with the cropping mesh of 5 voxels and further extraction of 64x64x64 sized boxes. Each subtomogram contained a piece of membrane and a fragment of a clathrin cage. Further, 3D-refinement and Dynamo classification resulted in 1702 particles. The same pipeline was applied to isolated endosomes, resulting in 27 particles.

For clathrin cages, obtained from primary hippocampal neurons, the same pipeline was applied. Due to the higher voxel size, the cropping mesh of 3 voxels was used, and further extraction of 60x60x60-sized boxes. Finally, 1564 particles were refined in Dynamo with a final resolution of 75.6 Å.

To measure CCVs radii, CCVs were picked along the membrane of the encapsulated vesicle using the surface model in the *Dynamo Catalogue* system[27] in at least three layers of the tomogram. The coordinates of the corresponding cropped positions were averaged to derive the vesicle center. Finally, sets of radii were calculated and averaged, to obtain radius estimation for each CCV.

To perform a single triskelia refinement both for CCVs from brain tissue and from primary hippocampal neurons, the following pipeline was applied: structures, obtained from the final CCVs classification round, were centered on the clathrin hexamer, with C6 symmetry applied. That made it possible to crop single triskelia. Further refinement of single-triskelion was

performed in Dynamo. The final amount of particles for cellular data was 3658 with a resolution of 81.1 Å (voxel size of 24.56 Å). The final number of triskelia particles that originated from brain-isolated CCV was 8006 (voxel size of 13.44 Å). Particles, containing triskelia from isolated mouse brain tissue, were imported in RELION-4.0[25] and further refined and classified, which led to the 15.6 Å resolution structure (voxel size of 3.36 Å).

Visualization and rendering of cryo-ET volumes

Volume rendering of subtomogram averages of V-ATPAse and CCV (main text), as well as clathrin coats in the Supplementary Figures, was prepared using ChimeraX[26]. Volume segmentation and rendering of SV surfaces with embedded V-ATPases (Fig. 1 *E*), CCV with a V-ATPase under the cage (Fig 3 *A*), and clathrin-coated endosomes (*SI Appendix, Fig. S9*) were prepared using AMIRA (Thermo Fisher Scientific and Zuse Institute Berlin). The raw volume slices of tomograms, subtomograms, and subtomogram averages were obtained using IMOD[28], Dynamo[24], or RELION-4. Schematic representation of used sample preparation procedures in Fig. 1 *A*, *C* and the SVs turnover scheme in Fig. 4 *F* are made with BioRender.com.

Negative staining of murine synaptic vesicle preparations

Synaptic vesicle preparations were diluted 1:100 (v/v) in PBS and 4 µL of the solution was applied to a glow-discharged continuous carbon grid. The sample was incubated on the grid for 30 s and washed with water for 30 s. Then, the grid was incubated with 2% uranyl-acetate for 30 s. The grids were imaged on a Zeiss 910, FEI Morgagni.

Proteinase K treatment of synaptic vesicles and proteolytic digestion

The PK-sensitivity assay was carried out as previously described[29]. In brief, synaptic vesicles were diluted in HB (without protease inhibitors) supplemented with 5 mM $CaCl_2$. In three replicates, vesicles were incubated with or without 50 µg/mL Proteinase K (PK) for 1 h at 37 °C. PK was inhibited with 5 mM PMSF for 10 min at RT. Proteins and polypeptides were reduced, alkylated, and digested using Lys-C (1:200 w/w) and trypsin (1:100 w/w). The digestion was stopped with 1% FA and peptides desalted with C18-StageTips. Proteomic samples for the determination of protein copy numbers and sample purity and composition were processed similarly but without the use of PK. The results of the described analysis can be found in *SI* Table S3.

LC-MS/MS analysis

Separation of the samples was achieved by reverse phase (RP)-HPLC on a Thermo Scientific™ Dionex™ UltiMate™ 3000 system connected to a PepMap C-18 trap column (Thermo Scientific) and an in-house packed C18 column for reversed-phase separation at 300 nL/min flow rate over120 or 180 min. Samples were analyzed on an Orbitrap Fusion or Orbitrap Fusion Lumos mass spectrometer with Instrument Control Software version 3.4. Data was acquired in data-dependent acquisition (DDA) mode. MS1 scans were acquired in the Orbitrap with a mass resolution of 120,000. MS1 scan range was set to m/z 375 – 1,500, 100% normalized AGC target, 50 ms maximum injection time, and 40 s dynamic exclusion. MS2 scans were acquired in the Ion trap in rapid mode. The normalized AGC target was set to 100%, 35 ms injection time, and an isolation window 1.6 m/z. Only precursors at charged states +2 to +4 were subjected to MS2. Peptides were fragmented using Normalized Collision Energy (NCE) of 30% or 32%.

Mass spectrometry data analysis

RAW files were searched using MaxQuant[30] (Version 2.0.3.0) using the following parameters: MS1 mass tolerance, 10 ppm; MS2 mass tolerance, 0.5 Da; the maximum number of missed

cleavages, 2; minimum peptide length, 7; peptide-mass, 500 – 5,000 Da. Carbamidomethylation (+ 57.021 Da) on cysteines was used as a static modification. Oxidation of methionine (+15.995 Da) and acetylation of the protein N-terminus (+42.011 Da) were set as a variable modification. Data were searched against the murine proteome retrieved from UniProt[31]. Label-free quantification[30] and iBAQ calculation were performed with a match between runs activated. Volcano plots were generated using the EnhancedVolcano v1.14.0 R package. Subcellular locations were retrieved from the Uniprot and transmembrane domains predicted using deepTMHMM[32]. The results can be found in *SI* Table S1.

Protein-protein interaction assay by LuTHy

Targeted validation of Syp interactions with LuTHy assays was performed as described in Trepte et al[3]. Briefly, open reading frames of candidate human SV proteins interactors (Syp, V-ATPase Voe1, V-ATPase Voa1, VAMP2) were cloned into LuTHy expression vectors (*SI* Table S4) by standard linear recombination reactions using the Gateway Cloning System and validated by restriction enzyme digests, agarose gel electrophoresis, and Sanger sequencing. All possible eight orientations were tested for interaction pairs: N/C-terminal tagging with Nano-luciferase (NL) or Protein A-mCitrine (PA-mCit). LuTHy control vectors expressing only NL or PA-mCit were used for the calculation of corrected scores. HEK293 cells were reverse transfected using linear polyethylenimine (25 kDa, Polysciences 23966) and LuTHy constructs; cells were subsequently incubated for 48 h. In-cell BRET measurements were carried out in flat-bottom white 96-well plates (Greiner, 655983) with 24 PPIs per plate (each PPI in triplicate). For cell-free LuC measurements, cells in 96-well plates were lysed and lysates were transferred to 384-well plates resulting in 96 PPIs per plate (Greiner, 784074). InfiniteÒ microplate readerM1000 (Tecan) was used for the readouts with the following settings: fluorescence of mCitrine recorded at Ex 500 nm/Em 530 nm, luminescence measured using blue (370–480 nm) and green (520–570 nm) bandpass filters with 1,000 ms (LuTHy-BRET) or 200 ms (LuTHy-LuC) integration time. A PPI was considered positive if its corrected BRET (cBRET) ratio was ≥ 0.03.

Characterization of synaptic vesicles and arrangement of V-ATPases

The identified SVs were characterized by their radii and the number of identified V-ATPases, observable (picked) on their surface. The described analysis was performed in MATLAB.

To estimate SV radii, the SV-containing subtomograms were cross-correlated with a set of binary vesicle-like masks of different radii. Each mask consisted of two encapsulated concentric spherical shells, imitating contrast produced by membrane leaflets. The cross-correlation allowed us to estimate the radii of SVs and to refine the coordinates of their centers.

Next, to calculate the number of V-ATPases and the pairwise distances between them, the subtomograms of the picked V-ATPases were first matched with the corresponding subtomograms of the SVs. The assignment was made based on the corresponding geometrical constraints using refined coordinates of vesicle centers and picked V-ATPases as well as ATPases orientations. For that, for each pair of SV and V-ATPase subtomogram, two vectors were constructed and compared:

- SV/V-ATPase vector - a vector from the refined vesicle center to the picked (presumably) V-ATPase position;
- V-ATPase vector - a unit vector representing the orientation of the V-ATPase in the membrane.

The SV/V-ATPase vector length was compared to the previously identified SV radius in order to reject picked particles that do not belong to a certain SV. Using the maximally allowed error of 5 voxels (voxel size = 13,44 Å), the V-ATPase particles were pre-assigned to the

corresponding synaptic vesicles. Further, SV/V-ATPase vector orientation was compared to the pre-assigned V-ATPase vector. If the absolute angular difference between the directions of those two vectors was not exceeding 5 degrees, the V-ATPase was finally associated with the current synaptic vesicle. Using the V-ATPases/SVs matching information, the correspondence between the number of observable V-ATPases per SV and the measured SV radius was further analyzed.

Models assembly for molecular complexes of V-ATPase and its proximal density candidates

To probe the suggested candidate proteins for the discovered density proximal to V-ATPase, we assembled their complexes with V-ATPase using the following procedure.

First, we used AlphaFold3 to obtain the predicted models for the complexes of each of eight candidates - Syp (UniProt: Q62277), Sypl1 (UniProt: O09117), Synpr (UniProt: Q8BGN8), Syngr1 (UniProt: O55100), Syngr3 (UniProt: Q8R191), SV2a (UniProt: Q9JIS5), SV2b (UniProt: Q8BG39), and SV2c (UniProt: Q69ZS6) - with V-ATPase Vo domain. For that we have used Voa1 (UniProt: Q9Z1G4), a brain-prevalent isoform Voe2 (UniProt: Q91XE7), and RNAseK (UniProt: Q8K3C0) V-ATPase domains, representing the Vo subcomplex near to the density of interest. The corresponding AF3 prediction quality metrics and predicted alignment error (PAE) plots are provided in the *SI* Table S7 and *SI Appendix*, Fig. S4 *B*, respectively. We also added ~200 PLM lipids to the AF3 prediction queries, since it increased the quality of the interface prediction between each candidate and the Vo subcomplex, measured by protein-only chain-to-all $ipTM_{prot}$ values of the candidate proteins (*SI* Table S7).

The reported protein-only chain-to-all $ipTM_{prot}$ is derived from the ipTM matrix for lipids-inclusive predictions, where all lipid-associated chain-to-chain ipTM values were excluded. That is due to the lipids-associated chain-to-chain ipTM values being extremely low (0.01-0.06), in line with the AF3 documentation. The AF3 guidelines note stricter TM scores (<0.05) for smaller molecules (including PLM lipids), which reduces the protein chain-to-all ipTM values of the lipids-inclusive predictions. At the same time, the suggested protein-only chain-to-all $ipTM_{prot}$ for lipids-inclusive predictions resemble greater protein-protein interface quality, than in the corresponding lipids-free predictions (see *SI* Table S7).

Next, we aimed to assemble the models of the candidates-V-ATPase complexes, considering the V-ATPase EM maps obtained via StA procedure. For that, we used the previously deposited purified human V-ATPase complex (PDB: 6wm2) and rigid-body fitted it (ChimeraX) to the obtained focused V-ATPase StA map from mouse brain tissue. After that, we removed lipid molecules from the AF3 predictions and rigid-body fit candidate-Vo complexes (ChimeraX) into the focused V-ATPase Vo map. To account for observed cryo-ET StA data, the candidate protein positions were further refined by sequential fit, available in ChimeraX. The respective refinement procedure included several steps: 1) the further unmovable V-ATPase model density was generated at the resolution of 22 Å (i.e. at the focused map resolution); 2) the generated V-ATPase density was subtracted from the focused StA map; 3) finally, each individual candidate protein model was rigid-body fitted into the obtained difference map. To ensure that re-fitted models are located within the respective distance error (PAE) of ~20-30 Å, reported by AF3 (see *SI Appendix*, Fig. S4 *B*), we calculated refinement shift RMSD for each candidate model: Syp - 10.8 Å, Synpr - 12.8 Å, Syngr1 - 16.0 Å, Syngr3 - 17.2 Å, Sypl1 - 14.4 Å, SV2a - 20.3 Å, SV2b - 10.5 Å and SV2c - 9.3 Å. Such an integrative refinement procedure allowed for the target complex model adjustment, accounting for the experimentally achieved StA map, while preserving the AF3 prediction information.

Thus, we predicted candidate-V-ATPase complexes by AF3, evaluated them, and re-assembled them with the full-V-ATPase model, considering the obtained experimental cryo-ET data. For better overall density representation, we combined the obtained global and focus-refined EM maps into a single one using available UCSF ChimeraX tools: *fitmap* - to superimpose initial global and focused maps, *vop maximum* - to merge them into a single fused map. The fused map with a full V-ATPase model and refined candidate density fits were used for the final representation (Fig. 2 *C, SI Appendix*, Fig. S4 *A*).

Finally, the candidate models were evaluated for their fit to the discovered density by the following scoring. First, the big enough binary mask for the target density was derived, covering the whole intracellular domain and transmembrane region of the density of interest. Next, this mask was applied to the experimental StA-focused map, resulting in the observed tight target density map, preserving its intracellular and transmembrane regions. Finally, we calculated the correlation for each candidate model at its refined position with the masked map of the discovered Vo-proximal density (see Table 1).

Simulation of randomly distributed V-ATPase pairs on the SV surface

To assess whether the observed distribution of the pairwise spherical distances between V-ATPases reflects their random allocation on the SV surface, the corresponding simulated dataset was generated. During those simulations, we took into account the orientation bias associated with the electron beam direction relative to particle orientations. The described simulation was performed in MATLAB.

The further described simulations utilized the following coordinate systems: Cartesian, spherical, and cylindrical. As a main XYZ frame, we decided to use a left-handed Cartesian coordinate system. For the other coordinate systems, the following conventions have been used:

a. spherical: the radial distance $r_{sph}\in[0;+\infty]$, azimuthal angle $\theta\in[0;2\pi]$ counted from axis X, and polar angle $\varphi\in[0;\pi]$ counted from axis Z of the XYZ frame;
b. cylindrical: radial distance $r_{cyl}\in[0;+\infty]$, azimuthal angle $\theta\in[0;2\pi]$ counted from axis X of the XYZ frame, and height $z\in[-\infty;+\infty]$.

First, to imitate a set of randomly allocated V-ATPase pairs on the SV surface, the points have to be sampled uniformly on the unit sphere surface. Note that independently sampling azimuthal angle θ from uniform distribution $U(0,\pi)$ and polar angle φ from $U(0,2\pi)$ would lead to the more dense arrangement of simulated points on the poles in comparison with the equator, making the resulting distribution non-uniform. In order to generate a uniform (equidistant) distribution of points on the sphere surface, we used the possibility of establishing a geometrical mapping between the sphere surface and the cylinder side surface (i.e. excluding its caps) circumscribed around that sphere. Thus, the target uniform distribution of the sphere surface points is achieved by generating their cylindrical coordinates - azimuthal angle θ from $U(0,\pi)$ and height z from $U(-1,1)$ - and mapping them back to the sphere surface.

Further, to consider the observed orientation bias of particle picking in the simulation, the corresponding orientation angles of the generated particles have to be sampled from the respective empirical distribution. To achieve that the following steps were performed:

1. The empirical distribution of spherical polar angles $\varphi_{emp}\in[0;\pi]$, representing orientation angles of particles relative to the beam direction, was mapped to the respective cylindrical height coordinates $z_{emp}\in[-1;1]$ using $z_{emp} = \cos(\varphi_{emp})$.
2. The corresponding empirical cumulative density function (eCDF) for the cylindrical height values z_{emp} distribution was generated, inverted ($eCDF^{-1}$) and equidistantly interpolated with the step of 0.01.

The obtained interpolation table of $eCDF^{-1}$ provides a mapping of uniformly generated numbers U(0,1) to the range of simulated cylindrical heights $z_{sim} \in [-1;1]$, allowing to account for the orientation bias while generating particle coordinates.

Finally, the target V-ATPases distribution was generated following the next procedure:

1. The simulated azimuthal angles $\theta_{sim} \in [0;2\pi]$ were sampled from the uniform distribution $U(0,2\pi)$.
2. The simulated cylindrical height values $z_{sim} \in [-1;1]$ were generated by mapping points sampled from U(0,1) using the obtained interpolation table of $eCDF^{-1}$.
3. To obtain the target simulated particle coordinates set, the generated cylindrical coordinates are transformed back to the spherical coordinates.

The described above approach allowed us to simulate the set of coordinates of V-ATPase pairs randomly allocated on the SV surface, taking into account the orientation bias. Based on the obtained pairs of particle coordinates, simulated on the unit sphere, the corresponding spherical (geodesic) distances between the V-ATPases were calculated and scaled according to the most abundant SV radius values of 19-20 nm. The number of simulated particle pairs was set to n=320, which is the number of experimentally observed vesicles containing only two ATPases within the described SV radius range.

Simulation of randomly distributed empty clathrin cages inside cells

To assess whether the observed distribution of the distances from empty clathrin cages to the closest synaptic membrane surfaces is shifted towards the synaptic membranes in the neuronal cells, the corresponding measurements on experimental data were conducted. To provide a reference, we also calculated the distance to the closest synaptic membrane surface from observed CCVs and SVs.

To account for a variety of observed neuronal cells in their size and shape, containing empty clathrin baskets, CCVs and SVs, we created 83 surface models by manual annotation of the cellular membranes in at least three tomogram layers using Dynamo Catalogue tools[27]. The obtained surface models were projected onto a 2D plane and the corresponding point clouds were thinned by calculating the moving-average positions of the sub-clouds of 10 dots sorted according to their polar angles. The obtained single-dot-width point curves were fitted using B-splines of the third order with 200 knots. The resulting set of enclosed curves served as the cell membrane models, reflecting the distribution of cells in size and shape.

Further, for each cell model, we measured the distances from empty clathrin baskets, CCVs and SVs to the closest point on the corresponding cell model curve.

Finally, all the experimental distributions of the distances from empty clathrin baskets, CCVs, and SVs to the closest synaptic membranes were pairwise compared using a two-sample Kolmogorov-Smirnov non-parametric test. The probed null hypothesis cumulative distribution function of the one experimental dataset was greater than that for the other experimental dataset. The obtained *p*-values for empty clathrin baskets versus SVs of 2.2e-4 << 0.05 and for CCVs versus SVs of 3.2e-8 << 0.05, suggest that the null hypothesis should be rejected in favor of the empty clathrin baskets and CCVs distances to membrane being both statistically significantly shifted towards smaller values in comparison to the SVs. Interestingly, the obtained *p*-values for empty clathrin baskets versus CCVs of 0.95 > 0.05, suggests that the shape of their distributions is indeed similar.

All the described calculations in this section were made using Python using open-source packages for data handling (pandas-2.0, numpy-1.25), statistical hypothesis testing (scipy-1.11), and planar geometry operations (shapely-2.0).

References for Supplementary Material

1. Abramson, J. *et al.* Accurate structure prediction of biomolecular interactions with AlphaFold 3. *Nature* 630, 493–500 (2024).
2. Wang, L., Wu, D., Robinson, C. V., Wu, H. & Fu, T.-M. Structures of a Complete Human V-ATPase Reveal Mechanisms of Its Assembly. *Mol. Cell* 80, 501-511.e3 (2020).
3. Trepte, P. *et al.* Lu TH y: a double-readout bioluminescence-based two-hybrid technology for quantitative mapping of protein–protein interactions in mammalian cells. *Mol. Syst. Biol.* 14, e8071 (2018).
4. Morris, K. L. *et al.* Cryo-EM of multiple cage architectures reveals a universal mode of clathrin self-assembly. *Nat. Struct. Mol. Biol.* 26, 890–898 (2019).
5. Takamori, S. *et al.* Molecular Anatomy of a Trafficking Organelle. *Cell* 127, 831–846 (2006).
6. Fernández-Chacón, R. *et al.* Synaptotagmin I functions as a calcium regulator of release probability. *Nature* 410, 41–49 (2001).
7. Fukuda, M., Aruga, J., Niinobe, M., Aimoto, S. & Mikoshiba, K. Inositol-1,3,4,5-tetrakisphosphate binding to C2B domain of IP4BP/synaptotagmin II. *J. Biol. Chem.* 269, 29206–29211 (1994).
8. Weingarten, D. J. *et al.* Fast resupply of synaptic vesicles requires synaptotagmin-3. *Nature* 611, 320–325 (2022).
9. Leenders, A. G. M., Da Silva, F. H. L., Ghijsen, W. E. J. M. & Verhage, M. Rab3A Is Involved in Transport of Synaptic Vesicles to the Active Zone in Mouse Brain Nerve Terminals. *Mol. Biol. Cell* 12, 3095–3102 (2001).
10. Huttner, W. B., Schiebler, W., Greengard, P. & De Camilli, P. Synapsin I (protein I), a nerve terminal-specific phosphoprotein. III. Its association with synaptic vesicles studied in a highly purified synaptic vesicle preparation. *J. Cell Biol.* 96, 1374–1388 (1983).
11. Medrihan, L. *et al.* Synapsin II desynchronizes neurotransmitter release at inhibitory synapses by interacting with presynaptic calcium channels. *Nat. Commun.* 4, 1512 (2013).
12. Li, G. *et al.* RalA and RalB Function as the Critical GTP Sensors for GTP-Dependent Exocytosis. *J. Neurosci.* 27, 190–202 (2007).
13. Sun, W., Tian, B., Wang, S., Liu, P. & Wang, Y. The function of SEC22B and its role in human diseases. *Cytoskeleton* 77, 303–312 (2020).
14. Fernández-Chacón, R., Achiriloaie, M., Janz, R., Albanesi, J. P. & Südhof, T. C. SCAMP1 Function in Endocytosis. *J. Biol. Chem.* 275, 12752–12756 (2000).
15. Falguières, T., Castle, D. & Gruenberg, J. Regulation of the MVB Pathway by SCAMP3. *Traffic* 13, 131–142 (2012).
16. Dong, M. *et al.* Glycosylated SV2A and SV2B Mediate the Entry of Botulinum Neurotoxin E into Neurons. *Mol. Biol. Cell* 19, 5226–5237 (2008).
17. Dunn, A. R. *et al.* Synaptic vesicle glycoprotein 2C (SV2C) modulates dopamine release and is disrupted in Parkinson disease. *Proc. Natl. Acad. Sci.* 114, (2017).
18. Kwon, S. E. & Chapman, E. R. Synaptophysin Regulates the Kinetics of Synaptic Vesicle Endocytosis in Central Neurons. *Neuron* 70, 847–854 (2011).
19. Janz, R. *et al.* Essential Roles in Synaptic Plasticity for Synaptogyrin I and Synaptophysin I. *Neuron* 24, 687–700 (1999).
20. McGeachan, R., Spires-Jones, M. P., Gilmore, M., Rose, J. & Spires-Jones, T. L. *Tau Accumulates in Synaptogyrin-3 Positive Synapses in Progressive Supranuclear Palsy*. http://medrxiv.org/lookup/doi/10.1101/2022.09.20.22280086 (2022) doi:10.1101/2022.09.20.22280086.
21. Knaus, P., Marquèze-Pouey, B., Scherer, H. & Betzt, H. Synaptoporin, a novel putative channel protein of synaptic vesicles. *Neuron* 5, 453–462 (1990).
22. Mossink, B. *et al.* Cadherin-13 is a critical regulator of GABAergic modulation in human stem-cell-derived neuronal networks. *Mol. Psychiatry* 27, 1–18 (2022).

23. Takeichi, M. CADHERINS: A MOLECULAR FAMILY IMPORTANT IN SELECTIVE CELL-CELL ADHESION. *Annu. Rev. Biochem.* 59, 237–252 (1990).
24. Castaño-Díez, D., Kudryashev, M., Arheit, M. & Stahlberg, H. Dynamo: A flexible, user-friendly development tool for subtomogram averaging of cryo-EM data in high-performance computing environments. *J. Struct. Biol.* 178, 139–151 (2012).
25. Zivanov, J. *et al.* A Bayesian approach to single-particle electron cryo-tomography in RELION-4.0. *eLife* 11, e83724 (2022).
26. Pettersen, E. F. *et al.* UCSF ChimeraX : Structure visualization for researchers, educators, and developers. *Protein Sci.* 30, 70–82 (2021).
27. Castaño-Díez, D., Kudryashev, M. & Stahlberg, H. Dynamo Catalogue: Geometrical tools and data management for particle picking in subtomogram averaging of cryo-electron tomograms. *J. Struct. Biol.* 197, 135–144 (2017).
28. Mastronarde, D. N. & Held, S. R. Automated tilt series alignment and tomographic reconstruction in IMOD. *J. Struct. Biol.* 197, 102–113 (2017).
29. Cvjetkovic, A. *et al.* Detailed Analysis of Protein Topology of Extracellular Vesicles–Evidence of Unconventional Membrane Protein Orientation. *Sci. Rep.* 6, 36338 (2016).
30. Cox, J. *et al.* Accurate Proteome-wide Label-free Quantification by Delayed Normalization and Maximal Peptide Ratio Extraction, Termed MaxLFQ. *Mol. Cell. Proteomics* 13, 2513–2526 (2014).
31. Magrane, M. & Consortium, U. UniProt Knowledgebase: a hub of integrated protein data. *Database* 2011, bar009–bar009 (2011).
32. Hallgren, J. *et al. DeepTMHMM Predicts Alpha and Beta Transmembrane Proteins Using Deep Neural Networks*. http://biorxiv.org/lookup/doi/10.1101/2022.04.08.487609 (2022) doi:10.1101/2022.04.08.487609.
